# Non-typhoidal *Salmonella* infections among children in a tertiary hospital in Ningbo, Zhejiang, China, 2012–2019

**DOI:** 10.1371/journal.pntd.0008732

**Published:** 2020-10-05

**Authors:** Yefang Ke, Wenbo Lu, Wenyuan Liu, Pan Zhu, Qunying Chen, Zhe Zhu

**Affiliations:** 1 Department of Clinical Laboratory, Ningbo Women and Children’s Hospital, Ningbo, Zhejiang, China; 2 Neonatal Intensive Care Unit, Ningbo Women and Children’s Hospital, Ningbo, Zhejiang, China; 3 Department of Blood Transfusion, HwaMei Hospital, University of Chinese Academy of Sciences, Ningbo, Zhejiang, China; 4 Ningbo Institute of Life and Health Industry, University of Chinese Academy of Sciences, Ningbo, Zhejiang, China; Pontificia Universidad Catolica de Chile, CHILE

## Abstract

**Background:**

Non-typhoidal *Salmonella* (NTS), a common cause of diarrheal enterocolitis, may also cause severe invasive diseases. Limited information on NTS infections in children is available in China.

**Methods:**

We performed a retrospective study of children admitted to the Ningbo Women and Children’s Hospital with culture-confirmed NTS infections between January 2012 and December 2019. Clinical and microbiological information were collected. We compared demographic, clinical and antibiotic resistance variables of invasive NTS (iNTS) infections and non-invasive NTS (non-iNTS) infections, and explored associations between hospitalizations for pediatric NTS infections and temperature and rainfall.

**Results:**

A total of 166 pediatric hospitalizations due to NTS infection were identified during the 8-year study period. Most of the 166 children were <5 years old (93.4%). The primary serotype was *Salmonella* Typhimurium (62.6%). Of 166 children with NTS infections, 11 had invasive infection. Compared to 155 children with non-iNTS infections, we found that iNTS infections were more likely to occur in infants ≤6 months or children with an underlying medical condition of leukemia at admission, but iNTS infections less often presented with a symptom of diarrhea (*P* <0.05 in all cases). The resistance rates of non-iNTS isolates to ceftazidime, ceftriaxone, cefepime, and aztreonam were significantly higher than those of iNTS isolates (*P* <0.05 in all cases). In addition, compared with iNTS isolates, non-iNTS isolates were significantly associated with resistance to ≥4 CLSI (Clinical and Laboratory Standard Institute) classes (*P* = 0.041, OR: 0.089, 95% CI: 0.009–0.901) and ≥2 first-line treatment agents (*P* = 0.040, OR: 0.159, 95% CI: 0.028–0.916). On the other hand, we found that seasonal NTS hospitalizations were positively associated with average seasonal temperature (r = 0.961, *P* = 0.039) and average monthly rainfall (r = 0.921, *P* <0.001).

**Conclusion:**

Non-iNTS accounts for the majority of infections in this study; infants ≤6 months and children with underlying medical conditions of leukemia are more likely to have invasive infection. The rates of antibiotic resistance in the iNTS isolates are generally lower than those in the non-iNTS isolates. On the other hand, high temperatures and heavy rainfall are positively associated with NTS hospitalizations among children in Ningbo.

## Author summary

Non-typhoidal *Salmonella* (NTS) infection is a foodborne disease with a global heavy burden. NTS usually causes diarrheal enterocolitis in humans and may also cause severe invasive NTS (iNTS) infections. Antimicrobial agents are not recommended for non-severe NTS diarrhea, but they are recommended for people at risk of severe or invasive infection. However, the recognition of iNTS infection among children is difficult before culture. We studied children who had NTS infections in a tertiary pediatric hospital in Ningbo and found that iNTS infections were more likely to occur in infants ≤6 months or children with an underlying medical condition of leukemia at admission, while diarrhea was more common in children with non-iNTS infections. The high rates of antibiotic resistance among children with NTS in Ningbo calls for continuous NTS surveillance. On the other hand, high temperatures and heavy rainfall were positively associated with NTS hospitalizations among children. These findings may help us to improve measures for the prevention, diagnosis and treatment of NTS infections among children.

## Introduction

Non-typhoidal *Salmonella* (NTS) infection, caused by *Salmonella enterica* subspecies *enterica* other than *Salmonella* Typhi and *Salmonella* Paratyphi, is a foodborne illness with a global heavy burden [1]. It is estimated that NTS causes 93.8 million gastroenteritis cases and 155,000 mortalities worldwide annually [1]. Additionally, invasive nontyphoidal *Salmonella* (iNTS) diseases have been established as one of the 19 additional diseases by the Global Burden of Diseases, Injuries, and Risk Factors Study (GBD) 2017 [2]. Previous studies have shown that children, especially young children, were the groups most susceptible to be NTS infected [3, 4]. A national study reported that 34% of diarrheal patients who were infected with NTS were aged <5 years old in China in 2008 [5]. A subsequent study in Guangdong Province in China showed a higher rate of infection among children aged <5 years, who accounted for 73% of the overall NTS infections from 2009 to 2012 [4].

The main manifestation of NTS infection is diarrheal enterocolitis, usually resulting from the ingestion of contaminated food [1]. However, NTS may also cause severe iNTS infections beyond the gastrointestinal tract, such as bacteremia, meningitis, and infections of other normally sterile sites. Children are susceptible to iNTS infection [6]. In sub-Saharan Africa, NTS is the leading cause of bacteremia, especially in infants, in children with malnutrition, anemia or malaria, and in individuals with HIV infection [7]. Studies have also shown a striking and underappreciated burden of iNTS disease in Asia [8, 9].

Moreover, antibiotic resistance and antimicrobial therapy are related to the invasiveness of NTS infections [10]. Some prior studies have found that NTS with higher rates of antibiotic resistance was more likely to cause invasive infections [11–13]; however, a recent study in the Netherlands reported that iNTS isolates were generally less (multi-) resistant [14]. The optimal choice and length of therapy for NTS infections in children are not clear [15, 16]. The diarrheal illness caused by NTS was usually self-limited; therefore, antimicrobial therapy is indicated only if it causes severe infections or invasive infection [15, 16]. Although there is increased recognition worldwide, little data on iNTS infection among children are available in China, and the characteristics of children who are more predisposed to invasive NTS disease and the corresponding antimicrobial therapy are less well studied.

Global warming brings significant public health problems [17, 18]. The variability in weather patterns and the increasingly warm temperatures have posed mounting threats to the distribution and spread of NTS infections [19, 20]. Some studies have shown significant associations between the incidence of NTS infections and meteorological variations, such as those observed in temperature, seasons, and rainfall [3, 21–24]. However, little is known about the associations between meteorological parameters and NTS infections among children in mainland China.

To better understand the epidemiology of NTS infections among children in Ningbo, Zhejiang Province, China and to provide information for formulating appropriate therapeutic and controlling strategies, we studied serotype distribution, antimicrobial resistance, and the clinical characteristics of NTS infections based on invasive and noninvasive status and investigated associations between temperature and rainfall and hospitalizations for pediatric NTS infections.

## Materials and methods

### Study site

This was a retrospective study conducted at Ningbo Women and Children’s Hospital, a 1200-bed tertiary hospital integrated with medical treatment, health care, teaching, scientific research, disease prevention, first aid and rehabilitation for women and children living in Ningbo. Ningbo, a 9816 km^2^ city in Zhejiang Province on the southeastern coast of China, has a total population of approximately 8,202,000. The city has a subtropical monsoon climate with four distinctive seasons. It is winter from December to February, spring from March to May, summer from June to August and autumn from September to November. Plum rain (the rainy season) and typhoons that bring heavy rainfall are common in the summer and early autumn.

### Data

Serotyped *Salmonella* isolates were searched from electronic medical record data in the Ningbo Women and Children’s Hospital from January 2012-December 2019. Available medical records were a unique identifier, sex, age, sampling date, clinical symptoms, underlying medical conditions, residence location, laboratory results, specimen type (stool, blood, cerebrospinal fluid, sputum, urine), bacterial antibiotic resistance and serotypes. Besides, the number of children who took stool culture for *Salmonella* during 2012–2019 was counted, repeated sampling was excluded, and NTS isolation rate from stool was calculated. Population data were obtained from the Ningbo 7^th^ census data 2018 of statistics (http://tjj.ningbo.gov.cn). Meteorological data were obtained from the Ningbo meteorological department (http://www.qx121.com). Agricultural data were obtained from the Ningbo Agricultural and Rural Bureau (http://www.nbnyj.gov.cn).

### Definitions

An NTS infection case in this study was defined as a child ≤14 years old residing in Ningbo with a culture-confirmed NTS infection during January 2012-December 2019. NTS infections can be divided into non-invasive NTS (non-iNTS) and invasive NTS (iNTS) infections based on different infection sites. The former denotes NTS isolated from stool, sputum, urine and vomiting, while the latter denotes NTS isolated from samples of blood, cerebrospinal fluid, or other normally sterile sites obtained within 48 h of admission [6].

A child could meet the iNTS case definition more than once if subsequent iNTS infection with the same serotype occurred more than 30 days after the index infection. A child could meet the non-iNTS case definition more than once if subsequent non-iNTS infection with the same serotype occurred more than 6 months after the index infection. NTS infection with the same serotype in both normally sterile sites and nonsterile sites <30 days from one another was defined as an iNTS infection case. Anemia was defined as a hemoglobin concentration <11.0 g/dL, and a hemoglobin concentration <5.0 g/dL was termed severe anemia. A respiratory tract infection case was defined as a child who had symptoms of respiratory tract infection with or without pulmonary imaging changes. The exclusive breastfeeding under 6 months was considered when an infant aged 0–5 months was fed exclusively with breast milk. Antibiotic resistance was defined as intermediate or resistant minimum inhibitory concentration. We determined the number of isolates that were fully susceptible and the number resistant to 1 class, 2 classes, 3 classes, and ≥4 classes. First-line treatment agents included the following: ampicillin, ceftriaxone, ciprofloxacin, and trimethoprim-sulfamethoxazole (TMP-SMX).

### Microbiological methods

The standardized indications for sampling were constant across the seasons. Stool were sampled when the patient had gastrointestinal symptoms. Cerebrospinal fluids (CSFs) were sampled when they had meningeal irritation signs or were suspected to be meningitis. Sputa were sampled when they had respiratory symptoms. Urine was sampled when they had urinary irritation symptoms or changes in routine urine tests or if urinary tract infections needed to be ruled out. Blood samples for culture were routinely performed in every child on admission in our hospital and were sampled again when they had symptoms of infection during hospitalization.

Briefly, stool samples were streaked and incubated on SS agar plates at 36°C for 18–48 h, and suspected colonies (characteristic colonies with black centers reflecting H_2_S production) were selected for inoculation on Kligler iron agar. CSF, sputum, and urine samples were cultured directly on blood agar at 36°C for 18–48 h. For CSF culturing, bacteria with a positive growth were isolated and cultured onto blood agar at 36°C for 18–24 h; for sputum culturing, bacteria with a growth advantage were isolated and cultured onto blood agar at 36°C for 18–24 h; for urine culturing, when fewer than three species of bacteria were grown, gram-negative bacteria with an estimated amount >10^5^ cfu/mL were isolated and cultured onto blood agar at 36°C for 18–24 h. For blood culturing, 1 mL to 3 mL of whole blood for bacterial culture was collected and inoculated into a pediatric bottle (BACT/ALERT PF, BioMérieux, France) and incubated in an automatic blood culture system (BACT/ALERT 3D) for 5 days or until rated positive. A positive blood culture was incubated on blood agar at 36°C for 18–24 h.

The identification and antibiotic susceptibility testing of NTS was performed using the VITEK 2 COMPACT automatic analysis system (BioMérieux, France), employing the GN and AST GN13 panels (BioMérieux, France) respectively. Standard strains (ATCC700323, ATCC25922, ATCC700327, ATCC29213) were used to control the microbiological quality. Isolates identified as belonging to *Salmonella* groups were further confirmed and serotyped by slide agglutination with commercial antisera (Ningbo Tianrun Bio-Pharmaceutical Co, Ltd, Ningbo, China). Serotypes with <5 isolates were combined into a category called *other*. The susceptibility breakpoints were those recommended by the Clinical and Laboratory Standards Institute guidelines (CLSI) for isolates obtained subsequently. The breakpoint criteria of the day in the analysis system were used to interpret the AST results, and the breakpoint criteria were updated in a timely manner when the CLSI guidelines changed. As AST GN13 has a detection limit of ciprofloxacin (0.25 μg/ml), intermediate inhibitory concentration to ciprofloxacin could not be evaluated below 0.25 μg/mL. To avoid duplication, consecutive culture samples and positive lab test results from the same patient were excluded.

### Statistical analysis

Clinical, laboratory and meteorology data were recorded in a Microsoft Excel database (Microsoft, Richmond, US). For continuous variables, normally distributed continuous data were presented as means ± standard deviations (SDs), and non-normally distributed variables were expressed as medians and interquartile ranges (IQRs); for categorical variables, data were presented as percentages. The comparison between iNTS and non-iNTS infections was calculated by logistic regression analysis; however, when zero observation was contained for a particular variable of one group, the *P* value was calculated with a two-tailed Fisher’s exact test instead. The Mann-Whitney test was used for the comparison of median age (non-normally distributed data) between iNTS and non-iNTS infections. Inter-annual linear trends of drug resistance were assessed by linear regression analysis. The numbers of NTS infection between every two seasons were compared using the chi-square test (categorical variables). The number of NTS infection cases and rainfall and temperature were examined by Spearman’s rank coefficient test. *P* <0.05 was considered statistically significant. All statistical analyses were performed using SPSS statistical 22.0 software (IBM, Armonk, NY, USA).

### Ethics statement

The study was approved by the Ningbo women and children’s hospital ethics committee (EC2020-007). All data analyzed were anonymized.

## Results

### Bacterial isolation and serotype distribution

**Fig 1:**
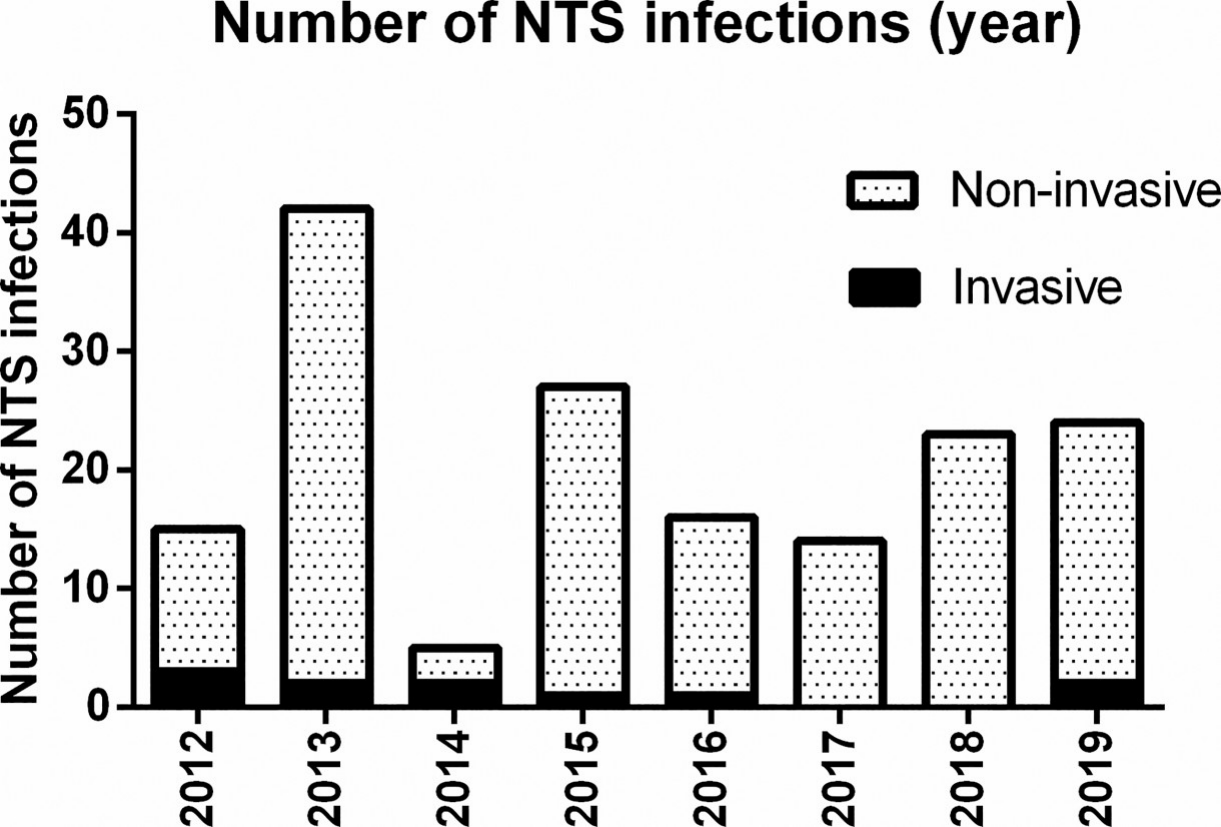
Distribution of the 166 NTS infection cases by year of admission and invasiveness. The bar chart shows the number of NTS infection cases hospitalized in Ningbo in each year from 2012 to 2019, according to the invasive or non-invasive status of infections. Abbreviations: NTS = non-typhoidal *Salmonella*.

During the 8-year study period, 166 children with NTS infections were identified (15 in 2012, 42 in 2013, 5 in 2014, 27 in 2015, 16 in 2016, 14 in 2017, 23 in 2018, 24 in 2019), 11 of whom had invasive infection and the remaining 155 of whom had non-iNTS (Fig 1).

As the most NTS isolates were detected in stool (91.6%, 152/166), an analysis of annual NTS isolation rates from stool was performed, which showed that isolation rates from stool were 0.90% in 2012, 1.97% in 2013, 0.08% in 2014, 2.32% in 2015, 1.24% in 2016, 0.93% in 2017, 1.29% in 2018, 1.04% in 2019 (S1 Fig).

All 166 children yielded a single serotype of NTS. Eighteen serotypes were identified among the 166 isolates. The primary serotype concerning the number of all NTS infection cases was Typhimurium (62.7%), followed by Dublin (7.8%), Enteritidis (4.8%), Choleraesuis (3.6%) and Bovis-morbificans (3.0%). Typhimurium was also the most common serotype in non-iNTS infection cases (Table 1).

**Table 1:** Distribution of 166 NTS isolates by invasive or non-invasive infection status and serotype, Ningbo, China, 2012–2019.

Serotype	No. (%) of isolates from NTS infections (n = 166)	No. (%) of isolates from iNTS infections (n = 11)	No. (%) of isolates from non-iNTS infections (n = 155)	Invasiveness^a^ (%)
Typhimurium	104 (62.6%)	2 (18.2%)	102 (65.8%)	1.9%
Dublin	13 (7.8%)	1 (9.1%)	12 (7.7%)	7.7%
Enteritidis	8 (4.8%)	1 (9.1%)	7 (4.5%)	12.5%
Choleraesuis	6 (3.6%)	1 (9.1%)	5 (3.2%)	16.7%
Bovis-morbificans	5 (3.0%)	1 (9.1%)	4 (2.6%)	20.0%
Untyped	5 (3.0%)	3 (27.3%)	2 (1.3%)	60.0%
*Other*	25 (15.1%)	2 (18.2%)	23 (14.8%)	8.0%

Abbreviations: NTS = non-typhoidal *Salmonella*, iNTS = invasive non-typhoidal *Salmonella*, non-iNTS = non-invasive non-typhoidal *Salmonella*

NTS with ≥5 isolates were listed individually, and those with <5 isolates were listed in the “*other*” category.

^a^ Proportion of iNTS infections to the total number of NTS infections in that serotype.

### Demographic, residential and clinical manifestations

One hundred (60.2%) of the 166 children with NTS infections were male. The median age of 166 children with NTS infection was 13 months (IQR: 8–21 months; range: 2 days-162 months). A total of 155 children (93.4%) were <5 years old. No child with malnutrition, malaria or HIV infection was reported. A total of 48 children (28.9%) had anemia (Hb <11.0 g/dL), but no severe anemia cases (Hb <5.0 g/dL) were reported. The most common anemia type was iron deficiency anemia (n = 44), 3 children was caused by leukemia, the last one was thalassaemia, while no child was suffered from hemolytic anemia in this study. Three of the 166 children had a medical history of leukemia, two of whom were aged >5 years old. Fever, diarrhea and bloody stools were the most common presenting features, noted in 143 (86.1%), 150 (90.4%) and 126 (75.90%) of the 166 children, respectively. Twenty children who had diarrhea were concomitantly infected with diarrheal virus (including 18 rotavirus, 1 adenovirus and 1 norovirus). We also investigated the feeding status in infants aged <6 months, and only 3 of 23 (13.0%) were exclusive breastfeeding, including a couple of 33-week premature twins who were readmitted within a month of birth.

Over the full study inclusion period, 117377 blood, 9586 urine, 29561 sputum, 6473 CSF and 12217 stool culture samples were screened in total. Most of the 155 non-iNTS infection cases had NTS isolated from stool (152, 98.1%), and of the remaining 3 cases, NTS was isolated from sputum in 2 and from urine in 1. One of the two children who had *Salmonella* isolated from sputum was a post term infant (42^+1^ weeks) admitting to hospital after 8 days of birth and the other one was a preterm infant (35^+2^ weeks) admitting to hospital after 22min of birth, they were all diagnosed as neonatal pneumonia; the child who had *Salmonella* isolated from urine was a 5-month boy, and was diagnosed as urinary tract infection. All eleven iNTS infection cases had NTS isolated from blood, of which three also had NTS isolated from cerebrospinal fluid. Two of the three children with culture-confirmed NTS meningitis were both 1 month of age, and the remaining child was aged 106 months.

We compared the demographics and clinical manifestations of iNTS infections with non-iNTS infections. Compared to 155 children with non-iNTS infections, we found that iNTS infections were more likely to occur in infants ≤6 months or children with underlying medical conditions of leukemia at admission but less often presented with a symptom of diarrhea, as revealed by bivariate and multivariable logistic regression analysis (*P* <0.05 in all cases) (Table 2).

**Table 2:** Demographic, residential and clinical manifestations of children with NTS infections, Ningbo, China, 2012–2019.

Characteristic	iNTS infections (n = 11)	Non-iNTS infections (n = 155)	Bivariate analysis		Multivariate analysis	
			*P*	OR (95%CI)	*P*	aOR (95%CI)
**Demographic**						
Male	7 (63.6%)	93 (60.0%)	0.812	1.167(0.328–4.154)		
Age, months (IQR)	13(6–93)	13(8–21)	0.915^a^	-		
Age ≤6 months	5 (45.5%)	27 (17.4%)	0.032	3.951(1.124–13.890)	0.040	4.508(1.069–19.016)
**Residence**						
Rural	3 (27.3%)	63 (40.6%)	0.387	0.548(0.140–2.144)		
**Investigations**						
Hb (mean ± SD)	11.6±1.6	11.8±1.3	0.737	0.924(0.583–1.465)		
Anemia (Hb <11.0 g/dL)	5 (45.5%)	43 (27.7%)	0.220	2.171(0.629–7.484)		
Severe anemia	0	0	-	-		
**Symptoms at admission**						
Fever	9 (81.8%)	134 (86.5%)	0.669	0.705(0.142–3.492)		
Diarrhea	6 (54.5%)	144 (92.9%)	<0.001	0.092(0.024–0.349)	0.020	0.170(0.038–0.760)
Bloody stools	6 (54.5%)	120 (77.4%)	0.098	0.350(0.101–1.216)		
Vomiting	6 (54.5%)	62 (40.0%)	0.349	1.800(0.526–6.155)		
Convulsion	2 (18.2%)	15 (9.7%)	0.378	2.074(0.410–10.502)		
**Co-morbidity**						
Respiratory tract infection	5 (45.5%)	46 (29.7%)	0.281	1.975(0.574–6.795)		
Gastrointestinal virus infection	1 (9.1%)	19 (12.3%)	0.756	0.716(0.087–5.910)		
Leukemia	2 (18.2%)	1 (0.6%)	0.005	34.222(2.829–413.913)	0.023	27.148(1.576–467.616)

All data of 166 children were available.

Abbreviations: NTS = non-typhoidal *Salmonella*, iNTS = invasive non-typhoidal *Salmonella*, non-iNTS = non-invasive non-typhoidal *Salmonella*, OR = odds ratio, aOR = adjusted odds ratio, 95%CI = 95% confidence interval

^a^ Mann-Whitney test was used to calculate *P* value (other variables: logistic regression)

- Not performed or predicts failure perfectly.

The routine therapeutic methods of NTS infections in our hospital were third-generation cephalosporins and piperacillin/sulbactam. All the children had recovered from NTS infections, and there was no subsequent case in the present study.

The details of demographic, isolation, serovar and clinical manifestations are presented in S1 Table.

### Antibiotic susceptibility

All 166 NTS isolates were tested for antimicrobial susceptibility. The overall rates of resistance to ampicillin, third-generation cephalosporins and ciprofloxacin were 78.3%, 34.9% and 19.3%, respectively. Besides, the antibiotic resistance profiles of the five main serotypes (Typhimurium, Dublin, Enteritidis, Choleraesuis, Bovis-morbificans) were analyzed, and found that the serotypes Typhimurium and Dublin had the greatest rate resistant to Ampicillin, Ampicillin/sulbactam, Ceftazidime, Ceftriaxone, Cefepime and Aztreonam; Typhimurium and Bovis-morbificans had greatest rate resistant to Ciprofloxacin and TMP-SMX (S2 Fig).

Eleven iNTS isolates were all susceptible to piperacillin/tazobactam, ceftazidime, ceftriaxone, cefepime, aztreonam, ertapenem, imipenem, and ciprofloxacin. The resistance rates of non-iNTS isolates to ceftazidime, ceftriaxone, cefepime, and aztreonam were significantly higher than those of iNTS isolates (*P* <0.05 in all cases) (Table 3).

**Table 3:** Antibiotic resistance among iNTS and non-iNTS infections, Ningbo, China, 2012–2019.

Antibiotic Resistance Type^a^	iNTS infections (n = 11)	Non-iNTS infections (n = 155)	*P*	OR (95%CI)
Agents				
Ampicillin	7 (63.6%)	123 (79.4%)	0.281	0.495(0.138–1.777)
Ampicillin/sulbactam	6 (54.5%)	115 (74.2%)	0.184	0.434(0.126–1.488)
Piperacillin/tazobactam	0 (0.0%)	10 (6.5%)	0.386^b^	-
Ceftazidime	0 (0.0%)	42 (27.1%)	0.046^b^	-
Ceftriaxone	0 (0.0%)	58 (37.4%)	0.012^b^	-
Cefepime	0 (0.0%)	43 (27.7%)	0.043^b^	-
Aztreonam	0 (0.0%)	49 (31.6%)	0.027^b^	-
Ertapenem	0 (0.0%)	2 (1.3%)	0.706^b^	-
Imipenem	0 (0.0%)	2 (1.3%)	0.706^b^	-
Ciprofloxacin	0 (0.0%)	32 (20.6%)	0.094^b^	-
Levofloxacin	1 (9.1%)	35 (22.6%)	0.314	0.343(0.043–2.757)
TMP-SMX	2 (18.2%)	48 (31.0%)	0.358	0.480(0.100–2.295)
Nitrofurantoin	4 (36.4%)	46 (29.7%)	0.654	1.337(0.375–4.760)
**Patterns**				
**Class level**^c^				
0 class (no resistant)	3(27.3%)	20(12.9%)	Reference	
1 class	2(18.2%)	12(7.7%)	0.915	1.111(0.162–7.632)
2 classes	1(9.1%)	21(13.5%)	0.337	0.317(0.030–3.311)
3 classes	4(36.4%)	27(17.4%)	0.988	0.988(0.198–4.915)
≥ 4 classes	1(9.1%)	75(48.3%)	0.041	0.089(0.009–0.901)
**First-line agents**^d^				
0 agent (no resistant)	4(36.4%)	27(17.4%)	Reference	
1 agent	5(45.5%)	43(27.7%)	0.735	0.785(0.194–3.183)
≥2 agents	2(18.2%)	85(54.8%)	0.040	0.159(0.028–0.916)

All antibiotic resistance data of 166 isolates were available

Abbreviations: NTS = non-typhoidal *Salmonella*, iNTS = invasive non-typhoidal *Salmonella*, non-iNTS = non-invasive non-typhoidal *Salmonella*, OR = odds ratio, CI = confidence interval

^a^ Resistant to single antimicrobials, 1 class, 2 classes, 3 classes, ≥4 classes and 1or more agents of first-line agents, resistance was defined as an intermediate or resistant minimum inhibitory concentration. (intermediate inhibitory concentration to ciprofloxacin, was evaluated as 0.25–0.5 μg/mL.)

^b^ Fisher exact test was used to calculate *P* value (other variables: logistic regression)

^c^ Based on 166 NTS isolates with complete information for all the tested antibiotics.

^d^ First-line agents: ampicillin, ceftriaxone, ciprofloxacin, or TMP-SMX.

*-* Predicts failure perfectly.

iNTS and non-iNTS isolates were further characterized according to susceptibility patterns, and non-iNTS isolates were also more likely to be multiresistant than iNTS isolates, as non-iNTS isolates were significantly associated with resistance to ≥ 4 CLSI classes (*P* = 0.041, OR: 0.089, 95% CI: 0.009–0.901) and ≥ 2 first-line treatment agents (*P* = 0.040, OR: 0.159, 95% CI: 0.028–0.916) (Table 3).

**Fig 2:**
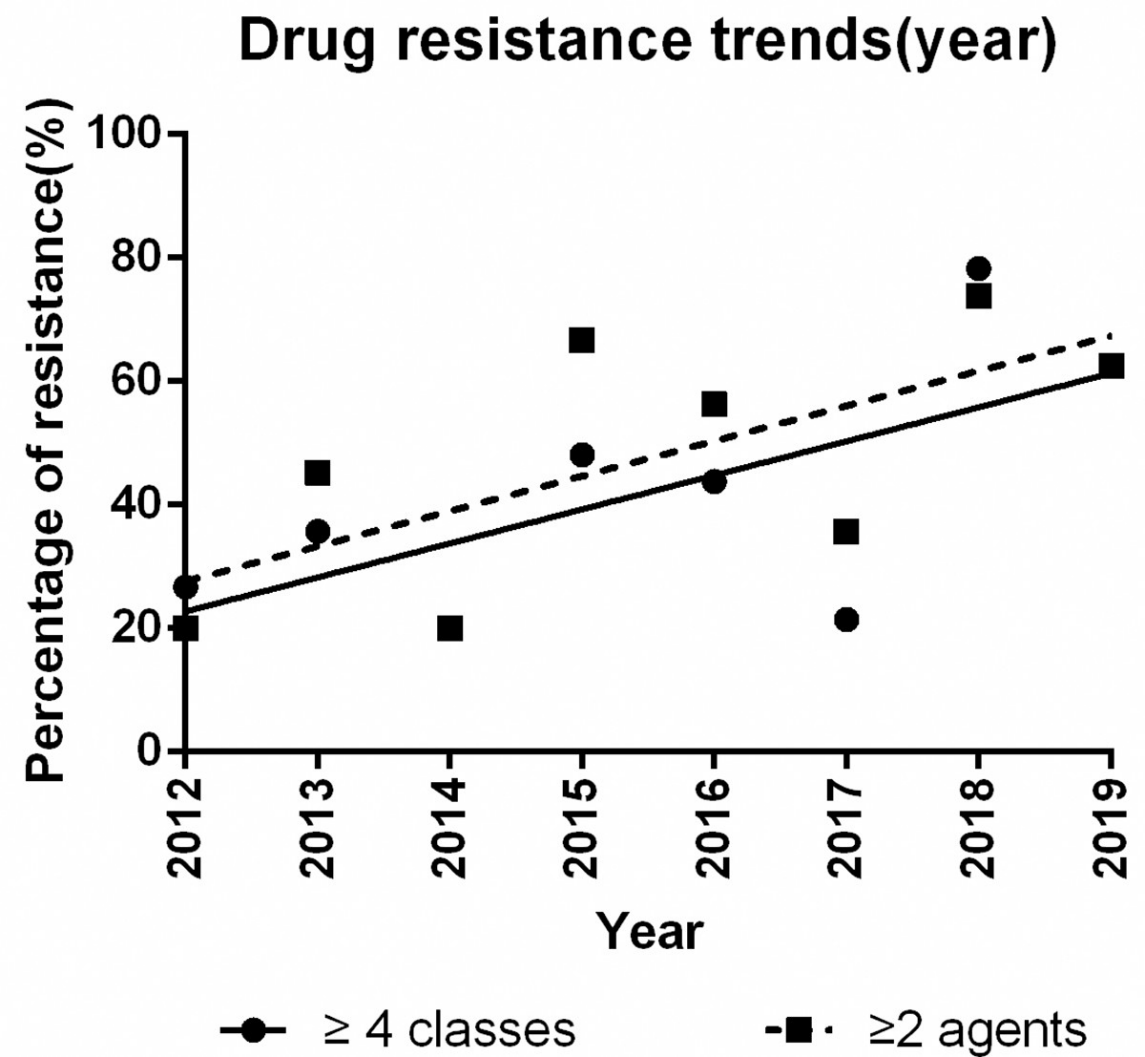
Drug resistance trends (resistant to≥ 4 CLSI classes and ≥2 first-line agents) of 166 *Salmonella* isolates in Ningbo,2012–2019. The percentage of resistance (%) over the 8-year study period in Ningbo is shown in the line graph.

The drug resistance trends (resistance to ≥ 4 CLSI classes and ≥ 2 first-line agents) from 2012 year to 2019 year were shown in Fig 2. There were upward trends in the both of resistance to ≥ 4 CLSI classes and ≥ 2 first-line agents over the years, with marginally significances were found (*P* = 0.076 and 0.069, respectively).

### Meteorological trends of NTS infection

**Fig 3:**
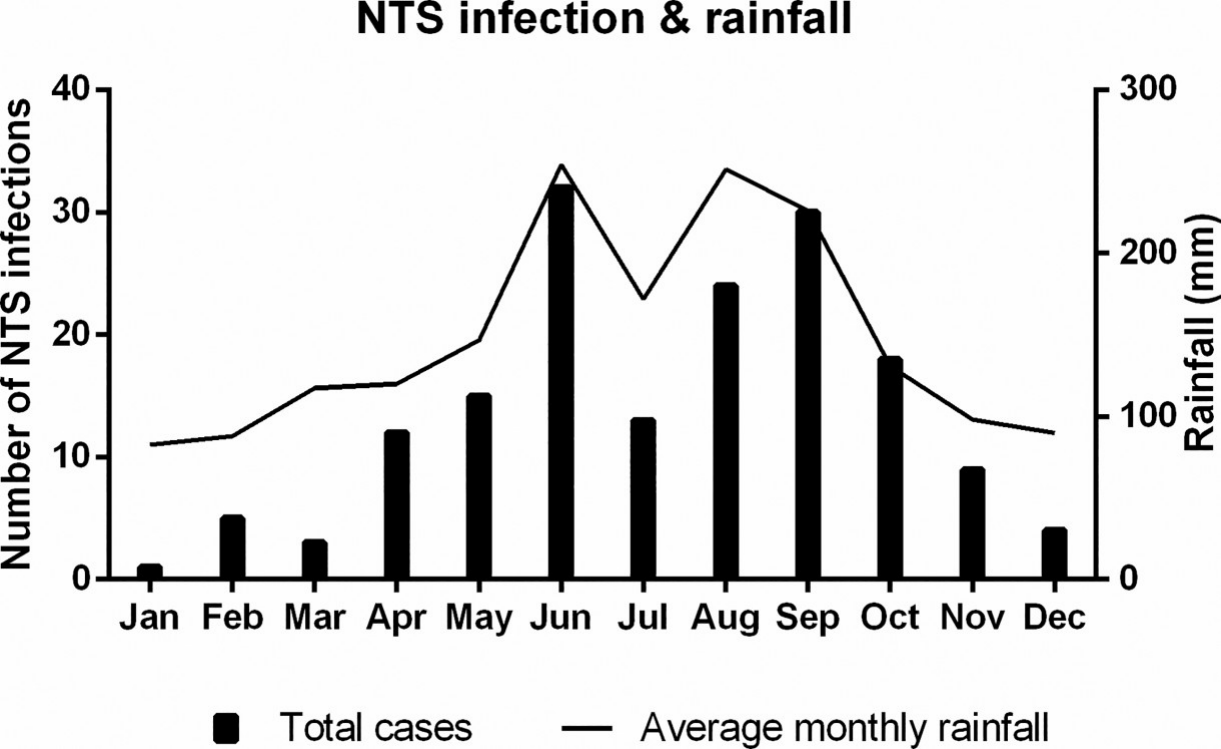
Distribution of 166 NTS infection cases and average monthly rainfall in Ningbo, 2012–2019. The bar chart shows the distribution of the 166 NTS infection cases according to the month of admission. The average monthly rainfall over the 8-year study period in Ningbo is shown in the line graph. Abbreviations: NTS = non-typhoidal *Salmonella*, Jan = January, Feb = February, Mar = March, Apr = April, Jun = June, Jul = July, Aug = August, Sep = September, Oct = October, Nov = November, Dec = December.

There was a positive association between monthly NTS hospitalizations and average monthly rainfall (r = 0.921, *P* <0.001) (Fig 3).

**Fig 4:**
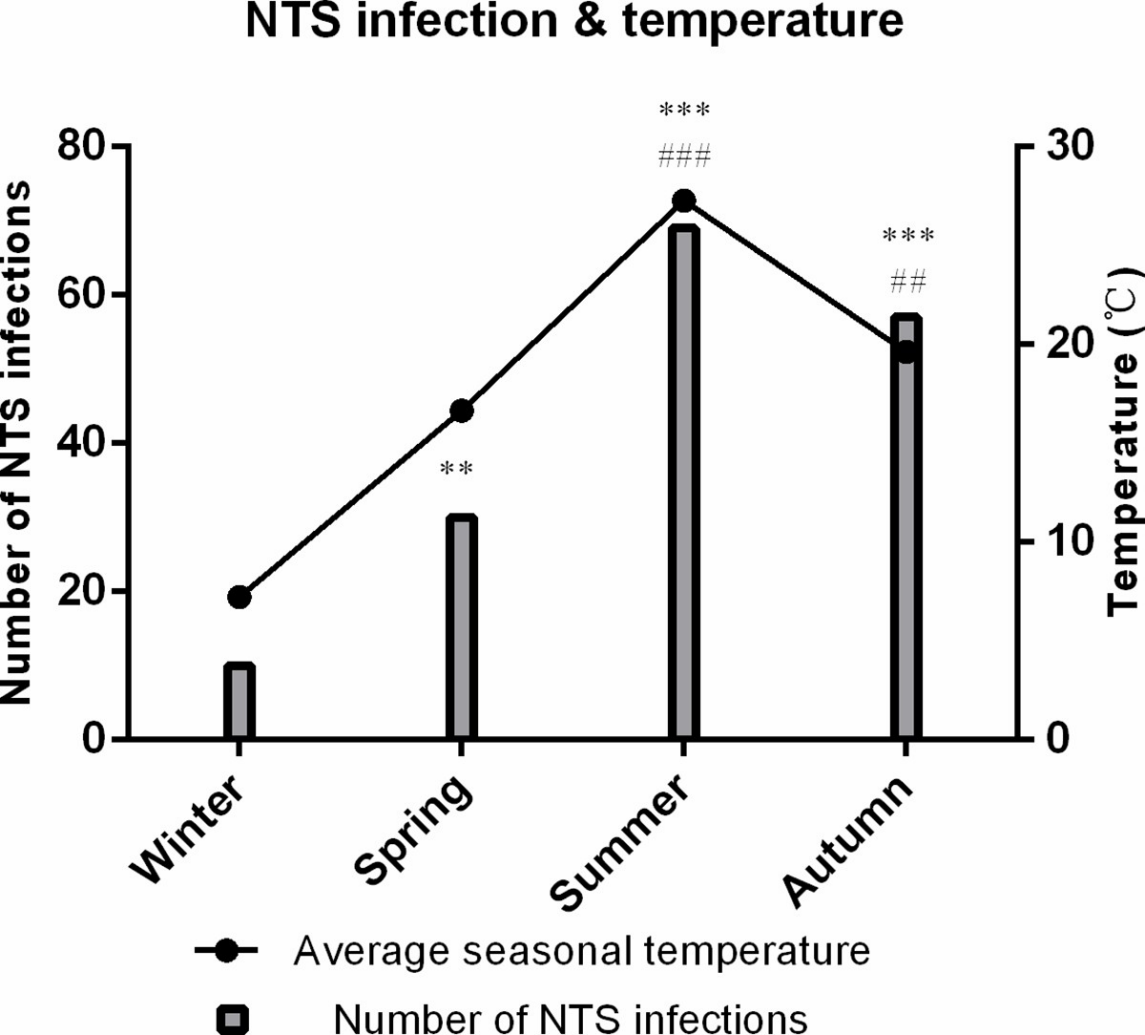
Distribution of the 166 NTS infection cases and average seasonal temperature in Ningbo, 2012–2019. The bar chart shows the distribution of the 166 NTS infection cases according to the admission. The average seasonal temperature over the 8-year study period in Ningbo is shown in the line graph. Difference in seasonal numbers of NTS infections: *P*<0.01 vs winter, *P*<0.001 vs winter, ## *P* <0.01 vs spring, ### *P* <0.001 vs spring, Abbreviations: NTS = non-typhoidal *Salmonella*.

The number of NTS infections were higher in summer and autumn than in winter and spring. It was also found that average seasonal temperature was positively associated with seasonal NTS hospitalizations (r = 0.961, *P* = 0.039) (Fig 4).

However, we did not observe any significant association between the number of iNTS cases and monthly rainfall (r = 0.167, *P* = 0.605)/seasonal temperature (r = 0.800, *P* = 0.200).

An analysis was performed to explore the relationships between NTS isolation rates from stool and monthly rainfall/seasonal temperature during 2012–2019. Just like the number of NTS isolated from all samples, NTS isolation rates from stool was both positively associated with monthly rainfall (r = 0.944, *P*<0.001) (S3 Fig), and seasonal temperature (r = 0.989, *P* = 0.011) (S4 Fig).

## Discussion

To our knowledge, this is the first detailed study of NTS infections among children in Ningbo, Zhejiang Province, China. There were 166 NTS infection cases in total, most of which were non-invasive infections (93.4%, 155/166). One hundred (60.2%) of the 166 children with NTS infections were males, and the median age of 166 children with NTS infection was 13 months (IQR: 8–21 months; range: 2 days-162 months). We found that age ≤6 months or suffering from an underlying medical condition of leukemia at admission were associated with iNTS infection, while diarrhea was more common in non-iNTS infections. Non-iNTS isolates were generally more (multi-) resistant than iNTS isolates were. Moreover, high temperatures and heavy rainfall were positively associated with NTS hospitalizations among children in Ningbo.

Children, especially those aged <5 years, were susceptible to *Salmonella* infection [4], and it was confirmed in our study that most of the children (93.4%) infected with NTS were younger than 5 years old. The main symptoms at admission were fever, diarrhea and bloody stools, while vomiting was less common in the present study. NTS disease is usually caused by commercially produced food contaminated by animal stool [10]. Outbreaks of *Salmonella* associated with infant milk products have been reported worldwide [25–28]. It has been confirmed that breastfeeding could decrease the risk of sporadic salmonellosis among infants, since exclusive breastfeeding could help infants avoid exposure to contaminated food [29]. Moreover, vitro studies also suggested that some components of human milk could inhibit the adhesion and invasiveness of *Salmonella* to human intestinal cells [30, 31]. In the present study, only 3 out of 23 infants (13%) aged <6 months were exclusively breastfeeding (including a set of 33-week premature twins who were readmitted within a month of birth), which was much lower than the national (29%) and global rate (41%) [32]. Further studies with case-control is needed to test whether exclusive breastfeeding could reduce the NTS infection in small babies.

NTS serotypes are diverse in their host range and epidemiology and vary in their propensity to cause invasive disease. We found that *Salmonella* Typhimurium was the most common serotype of NTS infection cases among children in Ningbo (62.7%), which was in accordance with the findings of studies performed in Guangzhou, southern China [4, 33, 34]. However, some studies from other regions of China [5, 35], the United States [11], the Netherlands [14], and Greece [36] reported that *Salmonella* Enteritidis was the most common serotype, followed by *Salmonella* Typhimurium. The modes of NTS transmission may be a key factor in the geographical variation in serotype distribution. It has been reported that *Salmonella* Typhimurium is considered to have a modest likelihood of causing iNTS infections, while other less common serotypes, such as Heidelberg, Choleraesuis, and Dublin, are more likely to cause invasive disease [10]. In our study, *Salmonella* Typhimurium was the primary serotype of non-iNTS, but there was no dominant serotype in iNTS infections.

The recognition of iNTS infections among children is difficult before culture because the clinical manifestation of iNTS infection is probably nonspecific [37]. Diarrhea was more often present in enterocolitis due to NTS infections, while iNTS infections can occur without diarrhea [16]. It was confirmed in our study with the incidence of diarrhea being significantly higher in the non-iNTS infection cases. In addition, some studies have reported that symptoms of pneumonia and respiratory distress occurred frequently in children with iNTS [9, 38, 39]. However, we did not find a significant difference in the morbidity of respiratory tract infection between iNTS cases and NTS cases. Previous studies have revealed that the risk factors for invasive disease in children included young age and immunosuppression of any cause [7, 39, 40], which were also revealed in the present study, as infants aged ≤6 months and children with the underlying medical condition of leukemia were prone to have iNTS infection.

An increasing prevalence of antimicrobial resistance has been observed in NTS over recent decades [41]. A substantial amount of resistance to antibiotics used for NTS infections was found in the present study, and accompanied with a upward trends over the years; 81.3% of isolates were resistant to at least one of the first-line agents, and more than half (52.4%) of isolates were resistant to two or more first-line agents. The development of resistance to extended-spectrum cephalosporins would be a challenge to empirical antimicrobial therapy among children, for whom fluoroquinolones should be avoided for their articular toxicity. Therefore, special emphasis should be placed on antimicrobial resistance. Especially in the present study, the overall resistance rates to third-generation cephalosporins (34.9%), ampicillin (78.3%), and ciprofloxacin (19.3%) were higher than those in previous domestic studies (11.7%-29.9%, 48.2%-76.6%, 5.5%-16.6%, respectively) [5, 33, 35]. This might be due to the overuse of antibiotics in animal husbandry and clinical practice, and additional, normal use might also lead to the selection of resistant bacteria.

A few studies have explored the health consequences of antimicrobial-resistant NTS, with conflicting results reported. The results that patients with antimicrobial-resistant NTS infection more likely to have invasive disease and severe outcomes were reported in the United States [11], while a study in the Netherlands found that iNTS isolates were generally less (multi-) resistant than non-iNTS isolates were [14]. Our results were similar to the latter. The relationship between antibiotic resistance and invasiveness of NTS has not yet been elucidated. It was reported that NTS could readily acquire antibiotic resistance mechanisms and evolve rapidly in response to antibiotic pressure [42]. However, virulence would be greatly influenced by the acquisition of resistance [43]. The fitness costs, including reduced growth rates, altered morphology, decreased motility, etc. were observed to be associated with the maintenance and expression of the ampC gene, and high-level ciprofloxacin resistance in *Salmonella* enterica isolates [44–47]. In summary, the consequence of the acquisition of resistance might related to the reduced expression of the invasion genes [48]. Further researches between antibiotic-resistant and invasive in non-typhoidal *Salmonella* is worthy of study.

NTS, as one of the most common zoonotic pathogens [49], can be attributed to animals such as layers, pigs, cattle, reptiles, and broilers [50, 51]. Animal-to-human transmission is recognized as the most important route of transmission in accounting for the current epidemiological patterns of NTS [16]. It was found that animal-borne *Salmonella* strains, which had the highest potential to cause human infections in China, were those that reside in chicken [52]. In Shanghai, *Salmonella* Typhimurium is frequently detected in raw food products and poultry stool [53]. The *Salmonella*-containing stool of live poultry might directly contaminate the meats that are sold together with live poultry via droplets, aerosol and dust [53]. We observed a decline in NTS infection in 2014, which may be due to a ban on live poultry trade in Ningbo and the reduction in poultry consumption, as the bird flu was endemic in China in 2014.

The patterns of NTS infections have been linked to temperature. In most prior studies, higher temperatures have been found to be associated with a higher incidence of NTS infection [3, 21–24]. This pattern was also reflected in hospitalized children with NTS infection in this study, in which the number of NTS infections was higher in the seasons with higher average temperature (r = 0.961, *P* = 0.039). This result was probably related to the fact that warm weather was beneficial for NTS colonization and growth in food and the environment and the virulence genes of NTS might be upregulated under high temperature condition [54, 55]. A previous study reported that the detection of *Salmonella* in animal stool was associated with season [56]. The high frequency and long distance of animal activities in the warmer season might cause them to have more exposure to sources of *Salmonella* in their environment or NTS-contaminated animal feed [57]. Meanwhile, a distinct seasonality in NTS of health significance was observed in the rural/agricultural streams, which is likely a reflection of seasonal source inputs in these watersheds [58]. On the other hand, human behavior might also play an important role, as people have more opportunity to eat food that is inadequately cooked and improperly stored during the warmer months.

Associations between NTS infections and rainfall have been less well studied. It has been found that rainfall could positively affect the detection and density of *Salmonella* in watersheds and produce [55, 59]. On the other hand, a humid environment caused by heavy rainfall has been reported to favor *Salmonella* proliferation and is positively associated with *Salmonella* hospitalizations in Hong Kong [3]. Typhoons and plum rain (the rainy season in June and July) often cause flooding and waterlogging disasters in Ningbo in the summer and early autumn and may contaminate agricultural land, surface water, crops or farmed animals, thus increasing the transmission of NTS.

However, some studies in Australia have reported paradoxical results in the relationship between rainfall and *Salmonella* detection, with a positive association reported in subtropical and tropical areas of Queensland [23], and a negative association was found in Adelaide, which is characterized by a typical Mediterranean climate [24]. Our founding in Ningbo supported the former. It is interesting to note that studies with positive associations being reported were all conducted in subtropical and tropical cities, where the climate was characterized by rainy and hot weather during the same period. In contrast, however, the Mediterranean climate is hot and dry in the summer and warm and humid in the winter, and rainfall and temperature are themselves negatively correlated. Therefore, it is difficult to separate the effects of temperature and precipitation. In our opinion, high temperature may be mainly helpful to the growth of NTS, while heavy rainfall plays a key role in the transmission of NTS.

As the most NTS isolates were detected in stool (91.6%, 152/166), an analysis of annual NTS isolation rates from stool was performed. The trends of NTS isolation rates from stool annually was almost paralleled to the number of NTS isolated, including it was lowest in 2014, when the bird flu was endemic in China. Besides, just like the number of NTS isolated from all samples, NTS isolation rates from stool was positively associated with monthly rainfall and seasonal temperature, which made the results more credible.

Eight-year meteorological data and a detailed hospitalization dataset of the largest pediatric hospital in Ningbo were adopted in the analysis, which enabled us to representatively evaluate the incidence and characteristics of severe local pediatric NTS infections. However, this study had some limitations. First, as we only used hospitalization data that represented severe infections, the true incidence of NTS infections might be underestimated. Second, in our study, the proportion of serotypes other than Typhimurium was much lower, and the analysis of the characteristics of the different serotypes was not fully performed, which might mask the role of serotypes. Third, tests of resistance genes and invasiveness genes, and MLST (multi-locus sequence typing) of NTS were not performed. Therefore, a study with a large sample size and relevant experiments is strongly encouraged. Fourth, because stool was sampled when the patient had gastrointestinal symptoms, seasonal variation of sampling might be related to the incidence of all-cause gastrointestinal infections. Fifth, because the detection limit of ciprofloxacin in AST GN13 (BioMérieux, France) is 0.25μg/mL. Therefore, it is impossible to detect the partial range of intermediate inhibitory concentration of ciprofloxacin (0.12–0.25μg/mL), which limits the analysis of more detail of ciprofloxacin AST results, and might differentiate the susceptibility to ciprofloxacin and levofloxacin in this study. Luckily, fluoroquinolones were rarely used in children with NTS infections.

## Conclusion

In this study, we found that most children infected with NTS were aged <5 years old, and the primary serotype was *Salmonella* Typhimurium. Additionally, iNTS accounts for the minority of infections; infants ≤6 months and children with underlying medical conditions of leukemia are more likely to have invasive infection. In addition, the rates of antibiotic resistance in the iNTS isolates are generally lower than those of non-iNTS isolates. On the other hand, hospitalizations for pediatric NTS infections are positively associated with seasonal temperature and monthly rainfall. Greater attention should be paid to the food safety of children to prevent the ingestion of *Salmonella*-contaminated food, particularly in the summer and autumn.

## Supporting information

S1 Fig Annual NTS isolation rates from stool. The bar chart shows the isolation rate of NTS isolates in each year from 2012 to 2019.
(TIF)

S2 Fig Antibiotic resistance of five main serotypes (Typhimurium, Dublin, Enteritidis, Choleraesuis, Bovis-morbificans) in Ningbo,2012–2019. The percentage of resistance (%) over the 8-year study period in Ningbo is shown in the line graph.
(TIF)

S3 Fig NTS isolation rates from stool and average monthly rainfall in Ningbo, 2012–2019. The bar chart shows the NTS isolation rates from stool according to the month. The average monthly rainfall over the 8-year study period in Ningbo is shown in the line graph. Abbreviations: NTS = non-typhoidal *Salmonella*, Jan = January, Feb = February, Mar = March, Apr = April, Jun = June, Jul = July, Aug = August, Sep = September, Oct = October, Nov = November, Dec = December.
(TIF)

S4 Fig NTS isolation rates from stool and average seasonal temperature in Ningbo, 2012–2019. The bar chart shows the NTS isolation rates from stool. The average seasonal temperature over the 8-year study period in Ningbo is shown in the line graph. Abbreviations: NTS = non-typhoidal *Salmonella*.
(TIF)

S1 Table Details of demographic, isolation date, residence, serovar and clinical manifestations for each strain. (DOCX)

## References

[1] Majowicz SE, Musto J, Scallan E, Angulo FJ, Kirk M, O'Brien SJ, et al The global burden of nontyphoidal *Salmonella* gastroenteritis. Clin Infect Dis. 2010; 50(6):882–889. Epub 2010/02/18. 10.1086/650733 . 20158401

[2] DALYs GBD, Collaborators H. Global, regional, and national disability-adjusted life-years (DALYs) for 359 diseases and injuries and healthy life expectancy (HALE) for 195 countries and territories, 1990–2017: a systematic analysis for the Global Burden of Disease Study 2017. Lancet. 2018; 392(10159):1859–1922. Epub 2018/11/13. 10.1016/S0140-6736(18)32335-3 . 30415748 PMC6252083

[3] Wang P, Goggins WB, Chan EYY. Associations of Salmonella hospitalizations with ambient temperature, humidity and rainfall in Hong Kong. Environ Int. 2018; 120:223–230. Epub 2018/08/14. 10.1016/j.envint.2018.08.014 . 30103121

[4] Liang Z, Ke B, Deng X, Liang J, Ran L, Lu L, et al Serotypes, seasonal trends, and antibiotic resistance of non-typhoidal *Salmonella* from human patients in Guangdong Province, China, 2009–2012. BMC Infect Dis. 2015; 15:53 Epub 2015/04/17. 10.1186/s12879-015-0784-4 . 25881319 PMC4343067

[5] Ran L, Wu S, Gao Y, Zhang X, Feng Z, Wang Z, et al Laboratory-based surveillance of nontyphoidal *Salmonella* infections in China. Foodborne Pathog Dis. 2011; 8(8):921–927. Epub 2011/04/16. 10.1089/fpd.2010.0827 . 21492026

[6] Parisi A, Crump JA, Stafford R, Glass K, Howden BP, Kirk MD. Increasing incidence of invasive nontyphoidal *Salmonella* infections in Queensland, Australia, 2007–2016. PLoS Negl Trop Dis. 2019; 13(3):e0007187 Epub 2019/03/19. 10.1371/journal.pntd.0007187 . 30883544 PMC6422252

[7] Uche IV, MacLennan CA, Saul A. A Systematic Review of the Incidence, Risk Factors and Case Fatality Rates of Invasive Nontyphoidal *Salmonella* (iNTS) Disease in Africa (1966 to 2014). PLoS Negl Trop Dis. 2017; 11(1):e0005118 Epub 2017/01/06. 10.1371/journal.pntd.0005118 . 28056035 PMC5215826

[8] Phu Huong Lan N, Le Thi Phuong T, Nguyen Huu H, Thuy L, Mather AE, Park SE, et al Invasive Non-typhoidal *Salmonella* Infections in Asia: Clinical Observations, Disease Outcome and Dominant Serovars from an Infectious Disease Hospital in Vietnam. PLoS Negl Trop Dis. 2016; 10(8):e0004857 Epub 2016/08/16. 10.1371/journal.pntd.0004857 . 27513951 PMC4981332

[9] Mohan A, Munusamy C, Tan YC, Muthuvelu S, Hashim R, Chien SL, et al Invasive *Salmonella* infections among children in Bintulu, Sarawak, Malaysian Borneo: a 6-year retrospective review. BMC Infect Dis. 2019; 19(1):330 Epub 2019/04/20. 10.1186/s12879-019-3963-x . 30999894 PMC6471830

[10] Crump JA, Sjolund-Karlsson M, Gordon MA, Parry CM. Epidemiology, Clinical Presentation, Laboratory Diagnosis, Antimicrobial Resistance, and Antimicrobial Management of Invasive *Salmonella* Infections. Clin Microbiol Rev. 2015; 28(4):901–937. Epub 2015/07/17. 10.1128/CMR.00002-15 . 26180063 PMC4503790

[11] Angelo K, Reynolds J, Karp B, Hoekstra R, Scheel CM, Friedman C. Antimicrobial Resistance Among Nontyphoidal *Salmonella* Isolated From Blood in the United States, 2003–2013. J Infect Dis. 2016; 214(10):1565–1570. Epub 2016/10/30. 10.1093/infdis/jiw415 . 27609807 PMC6668032

[12] Krueger AL, Greene SA, Barzilay EJ, Henao O, Vugia D, Hanna S, et al Clinical outcomes of nalidixic acid, ceftriaxone, and multidrug-resistant nontyphoidal *Salmonella* infections compared with pansusceptible infections in FoodNet sites, 2006–2008. Foodborne Pathog Dis. 2014; 11(5):335–341. Epub 2014/03/13. 10.1089/fpd.2013.1642 . 24617446

[13] Crump JA, Medalla FM, Joyce KW, Krueger AL, Hoekstra RM, Whichard JM, et al Antimicrobial resistance among invasive nontyphoidal *Salmonella* enterica isolates in the United States: National Antimicrobial Resistance Monitoring System, 1996 to 2007. Antimicrob Agents Chemother. 2011; 55(3):1148–1154. Epub 2011/01/05. 10.1128/AAC.01333-10 . 21199924 PMC3067073

[14] Mughini-Gras L, Pijnacker R, Duijster J, Heck M, Wit B, Veldman K, et al Changing epidemiology of invasive non-typhoid *Salmonella* infection: a nationwide population-based registry study. Clin Microbiol Infect. 2019 Epub 2019/11/25. 10.1016/j.cmi.2019.11.015 . 31760114

[15] Wen SC, Best E, Nourse C. Non-typhoidal *Salmonella* infections in children: Review of literature and recommendations for management. J Paediatr Child Health. 2017; 53(10):936–941. Epub 2017/05/31. 10.1111/jpc.13585 . 28556448

[16] Onwuezobe IA, Oshun PO, Odigwe CC. Antimicrobials for treating symptomatic non-typhoidal *Salmonella* infection. Cochrane Database Syst Rev. 2012; 11:CD001167 Epub 2012/11/16. 10.1002/14651858.CD001167.pub2 . 23152205 PMC6532567

[17] Patz JA. Global warming. BMJ. 2004; 328(7451):1269–1270. Epub 2004/05/29. 10.1136/bmj.328.7451.1269 . 15166039 PMC420153

[18] Balakrishnan VS. Global warming: experts demand urgent action to prevent public health crisis. BMJ. 2018; 363:k4241 Epub 2018/10/12. 10.1136/bmj.k4241 . 30301717

[19] Milazzo A, Giles LC, Zhang Y, Koehler AP, Hiller JE, Bi P. Heatwaves differentially affect risk of *Salmonella* serotypes. J Infect. 2016; 73(3):231–240. Epub 2016/06/19. 10.1016/j.jinf.2016.04.034 . 27317378

[20] Jiang C, Shaw KS, Upperman CR, Blythe D, Mitchell C, Murtugudde R, et al Climate change, extreme events and increased risk of salmonellosis in Maryland, USA: Evidence for coastal vulnerability. Environ Int. 2015; 83:58–62. Epub 2015/06/22. 10.1016/j.envint.2015.06.006 . 26093493 PMC6590700

[21] Milazzo A, Giles LC, Zhang Y, Koehler AP, Hiller JE, Bi P. The effect of temperature on different *Salmonella* serotypes during warm seasons in a Mediterranean climate city, Adelaide, Australia. Epidemiol Infect. 2016; 144(6):1231–1240. Epub 2015/11/03. 10.1017/S0950268815002587 . 26522685

[22] Grjibovski AM, Kosbayeva A, Menne B. The effect of ambient air temperature and precipitation on monthly counts of salmonellosis in four regions of Kazakhstan, Central Asia, in 2000–2010. Epidemiol Infect. 2014; 142(3):608–615. Epub 2013/07/03. 10.1017/S095026881300157X . 23816177 PMC9151133

[23] Zhang Y, Bi P, Hiller JE. Climate variations and *Salmonella* infection in Australian subtropical and tropical regions. Sci Total Environ. 2010; 408(3):524–530. Epub 2009/11/20. 10.1016/j.scitotenv.2009.10.068 . 19922981

[24] Zhang Y, Bi P, Hiller J. Climate variations and salmonellosis transmission in Adelaide, South Australia: a comparison between regression models. Int J Biometeorol. 2008; 52(3):179–187. Epub 2007/07/12. 10.1007/s00484-007-0109-4 . 17623111

[25] Jourdan-da Silva N, Fabre L, Robinson E, Fournet N, Nisavanh A, Bruyand M, et al Ongoing nationwide outbreak of *Salmonella* Agona associated with internationally distributed infant milk products, France, December 2017. Euro Surveill. 2018; 23(2). Epub 2018/01/18. 10.2807/1560-7917.ES.2018.23.2.17-00852 . 29338811 PMC5770849

[26] Jones G, Pardos de la Gandara M, Herrera-Leon L, Herrera-Leon S, Varela Martinez C, Hureaux-Roy R, et al Outbreak of *Salmonella* enterica serotype Poona in infants linked to persistent *Salmonella* contamination in an infant formula manufacturing facility, France, August 2018 to February 2019. Euro Surveill. 2019; 24(13). Epub 2019/04/04. 10.2807/1560-7917.ES.2019.24.13.1900161 . 30940315 PMC6446512

[27] Cahill SM, Wachsmuth IK, Costarrica Mde L, Ben Embarek PK. Powdered infant formula as a source of *Salmonella* infection in infants. Clin Infect Dis. 2008; 46(2):268–273. Epub 2008/01/04. 10.1086/524737 . 18171262

[28] Yang B, Zhao H, Cui S, Wang Y, Xia X, Xi M, et al Prevalence and characterization of *Salmonella* enterica in dried milk-related infant foods in Shaanxi, China. J Dairy Sci. 2014; 97(11):6754–6760. Epub 2014/09/15. 10.3168/jds.2014-8292 . 25218754

[29] Rowe SY, Rocourt JR, Shiferaw B, Kassenborg HD, Segler SD, Marcus R, et al Breast-feeding decreases the risk of sporadic salmonellosis among infants in FoodNet sites. Clin Infect Dis. 2004; 38 Suppl 3:S262–270. Epub 2004/04/20. 10.1086/381595 . 15095198

[30] Coppa GV, Facinelli B, Magi G, Marini E, Zampini L, Mantovani V, et al Human milk glycosaminoglycans inhibit in vitro the adhesion of Escherichia coli and *Salmonella* fyris to human intestinal cells. Pediatr Res. 2016; 79(4):603–607. Epub 2015/12/19. 10.1038/pr.2015.262 . 26679156

[31] Liu B, Yu Z, Chen C, Kling DE, Newburg DS. Human milk mucin 1 and mucin 4 inhibit *Salmonella* enterica serovar Typhimurium invasion of human intestinal epithelial cells in vitro. J Nutr. 2012; 142(8):1504–1509. Epub 2012/06/22. 10.3945/jn.111.155614 . 22718031 PMC3397338

[32] Wang Y, Zhou C. China should take more measures to raise its breastfeeding rate. Biosci Trends. 2019; 13(4):358–360. Epub 2019/09/19. 10.5582/bst.2019.01240 . 31527332

[33] Liang B, Xie Y, He S, Mai J, Huang Y, Yang L, et al Prevalence, serotypes, and drug resistance of nontyphoidal *Salmonella* among paediatric patients in a tertiary hospital in Guangzhou, China, 2014–2016. J Infect Public Health. 2019; 12(2):252–257. Epub 2018/11/24. 10.1016/j.jiph.2018.10.012 . 30466903

[34] Ren L, Yang M, Geng L, Chen P, Chen H, Gong S, et al Nontyphoidal *Salmonella* Gastroenteritis in a Tertiary Children's Hospital in Southern China: Characteristics and Dietary Considerations. Gastroenterol Res Pract. 2018; 2018:3097468 Epub 2018/04/25. 10.1155/2018/3097468 . 29686701 PMC5857331

[35] Li Y, Xie X, Xu X, Wang X, Chang H, Wang C, et al Nontyphoidal *Salmonella* infection in children with acute gastroenteritis: prevalence, serotypes, and antimicrobial resistance in Shanghai, China. Foodborne Pathog Dis. 2014; 11(3):200–206. Epub 2013/12/10. 10.1089/fpd.2013.1629 . 24313784

[36] Galanakis E, Bitsori M, Maraki S, Giannakopoulou C, Samonis G, Tselentis Y. Invasive non-typhoidal salmonellosis in immunocompetent infants and children. Int J Infect Dis. 2007; 11(1):36–39. Epub 2006/03/28. 10.1016/j.ijid.2005.09.004 . 16564718

[37] Crump JA, Heyderman RS. A Perspective on Invasive *Salmonella* Disease in Africa. Clin Infect Dis. 2015; 61 Suppl 4:S235–240. Epub 2015/10/10. 10.1093/cid/civ709 . 26449937 PMC4596931

[38] Schwarz NG, Sarpong N, Hunger F, Marks F, Acquah SE, Agyekum A, et al Systemic bacteraemia in children presenting with clinical pneumonia and the impact of non-typhoid *Salmonella* (NTS). BMC Infect Dis. 2010; 10:319 Epub 2010/11/06. 10.1186/1471-2334-10-319 . 21050455 PMC2991321

[39] MacLennan CA, Msefula CL, Gondwe EN, Gilchrist JJ, Pensulo P, Mandala WL, et al Presentation of life-threatening invasive nontyphoidal *Salmonella* disease in Malawian children: A prospective observational study. PLoS Negl Trop Dis. 2017; 11(12):e0006027 Epub 2017/12/08. 10.1371/journal.pntd.0006027 . 29216183 PMC5745124

[40] Muthumbi E, Morpeth SC, Ooko M, Mwanzu A, Mwarumba S, Mturi N, et al Invasive Salmonellosis in Kilifi, Kenya. Clin Infect Dis. 2015; 61 Suppl 4:S290–301. Epub 2015/10/10. 10.1093/cid/civ737 . 26449944 PMC4596936

[41] Zhan Z, Xu X, Gu Z, Meng J, Wufuer X, Wang M, et al Molecular epidemiology and antimicrobial resistance of invasive non-typhoidal *Salmonella* in China, 2007–2016. Infect Drug Resist. 2019; 12:2885–2897. Epub 2019/10/02. 10.2147/IDR.S210961 . 31571942 PMC6750164

[42] Peng M, Salaheen S, Buchanan RL, Biswas D. Alterations of *Salmonella* enterica Serovar Typhimurium Antibiotic Resistance under Environmental Pressure. Appl Environ Microbiol. 2018; 84(19). Epub 2018/07/29. 10.1128/AEM.01173-18 . 30054356 PMC6146977

[43] Beceiro A, Tomas M, Bou G. Antimicrobial resistance and virulence: a successful or deleterious association in the bacterial world? Clin Microbiol Rev. 2013; 26(2):185–230. Epub 2013/04/05. 10.1128/CMR.00059-12 . 23554414 PMC3623377

[44] O'Regan E, Quinn T, Frye JG, Pages JM, Porwollik S, Fedorka-Cray PJ, et al Fitness costs and stability of a high-level ciprofloxacin resistance phenotype in *Salmonella* enterica serotype enteritidis: reduced infectivity associated with decreased expression of *Salmonella* pathogenicity island 1 genes. Antimicrob Agents Chemother. 2010; 54(1):367–374. Epub 2009/11/18. 10.1128/AAC.00801-09 . 19917752 PMC2798541

[45] Wang YP, Li L, Shen JZ, Yang FJ, Wu YN. Quinolone-resistance in *Salmonella* is associated with decreased mRNA expression of virulence genes invA and avrA, growth and intracellular invasion and survival. Vet Microbiol. 2009; 133(4):328–334. Epub 2008/09/03. 10.1016/j.vetmic.2008.07.012 . 18762392

[46] Fabrega A, du Merle L, Le Bouguenec C, Jimenez de Anta MT, Vila J. Repression of invasion genes and decreased invasion in a high-level fluoroquinolone-resistant *Salmonella* typhimurium mutant. PLoS One. 2009; 4(11):e8029 Epub 2009/12/01. 10.1371/journal.pone.0008029 . 19946377 PMC2777507

[47] Morosini MI, Ayala JA, Baquero F, Martinez JL, Blazquez J. Biological cost of AmpC production for *Salmonella* enterica serotype Typhimurium. Antimicrob Agents Chemother. 2000; 44(11):3137–3143. Epub 2000/10/19. 10.1128/aac.44.11.3137-3143.2000 . 11036037 PMC101617

[48] Fabrega A, Vila J. *Salmonella* enterica serovar Typhimurium skills to succeed in the host: virulence and regulation. Clin Microbiol Rev. 2013; 26(2):308–341. Epub 2013/04/05. 10.1128/CMR.00066-12 . 23554419 PMC3623383

[49] Eurosurveillance editorial t. The 2013 joint ECDC/EFSA report on trends and sources of zoonoses, zoonotic agents and food-borne outbreaks published. Euro Surveill. 2015; 20(4). Epub 2015/02/07. 10.2807/ese.20.04.21021-en . 25655057

[50] Mughini-Gras L, Enserink R, Friesema I, Heck M, van Duynhoven Y, van Pelt W. Risk factors for human salmonellosis originating from pigs, cattle, broiler chickens and egg laying hens: a combined case-control and source attribution analysis. PLoS One. 2014; 9(2):e87933 Epub 2014/02/08. 10.1371/journal.pone.0087933 . 24503703 PMC3913680

[51] Mughini-Gras L, Heck M, van Pelt W. Increase in reptile-associated human salmonellosis and shift toward adulthood in the age groups at risk, the Netherlands, 1985 to 2014. Euro Surveill. 2016; 21(34). Epub 2016/09/03. 10.2807/1560-7917.ES.2016.21.34.30324 . 27589037 PMC5144934

[52] Wu C, Yan M, Liu L, Lai J, Chan EW, Chen S. Comparative characterization of nontyphoidal *Salmonella* isolated from humans and food animals in China, 2003–2011. Heliyon. 2018; 4(4):e00613 Epub 2018/05/08. 10.1016/j.heliyon.2018.e00613 . 29736431 PMC5934692

[53] Yang X, Jin K, Yang F, Yuan G, Liu W, Xiang L, et al Nontyphoidal *Salmonella* Gastroenteritis in Baoshan, Shanghai, China, 2010 to 2014: An Etiological Surveillance and Case-Control Study. J Food Prot. 2017; 80(3):482–487. Epub 2017/02/17. 10.4315/0362-028X.JFP-16-309 . 28207307

[54] Yang Y, Khoo WJ, Zheng Q, Chung HJ, Yuk HG. Growth temperature alters *Salmonella* Enteritidis heat/acid resistance, membrane lipid composition and stress/virulence related gene expression. Int J Food Microbiol. 2014; 172:102–109. Epub 2013/12/26. 10.1016/j.ijfoodmicro.2013.12.006 . 24368153

[55] Strawn LK, Fortes ED, Bihn EA, Nightingale KK, Grohn YT, Worobo RW, et al Landscape and meteorological factors affecting prevalence of three food-borne pathogens in fruit and vegetable farms. Appl Environ Microbiol. 2013; 79(2):588–600. Epub 2012/11/13. 10.1128/AEM.02491-12 . 23144137 PMC3553790

[56] Bondo KJ, Pearl DL, Janecko N, Boerlin P, Reid-Smith RJ, Parmley J, et al Impact of Season, Demographic and Environmental Factors on *Salmonella* Occurrence in Raccoons (Procyon lotor) from Swine Farms and Conservation Areas in Southern Ontario. PLoS One. 2016; 11(9):e0161497 Epub 2016/09/10. 10.1371/journal.pone.0161497 . 27611198 PMC5017689

[57] Crump JA, Griffin PM, Angulo FJ. Bacterial contamination of animal feed and its relationship to human foodborne illness. Clin Infect Dis. 2002; 35(7):859–865. Epub 2002/09/14. 10.1086/342885 . 12228823

[58] Thomas JL, Slawson RM, Taylor WD. *Salmonella* serotype diversity and seasonality in urban and rural streams. J Appl Microbiol. 2013; 114(3):907–922. Epub 2012/11/22. 10.1111/jam.12079 . 23167768

[59] Haley BJ, Cole DJ, Lipp EK. Distribution, diversity, and seasonality of waterborne *Salmonella*e in a rural watershed. Appl Environ Microbiol. 2009; 75(5):1248–1255. Epub 2009/01/07. 10.1128/AEM.01648-08 . 19124594 PMC2648171

