# Supplementary figures and images for: Non-typhoidal Salmonella infections among children in a tertiary hospital in Ningbo, Zhejiang, China, 2012–2019
Source: PLoS Negl Trop Dis. 2020 Oct 5;14(10):e0008732. doi: 10.1371/journal.pntd.0008732 (PMC7561262; doi:10.1371/journal.pntd.0008732)

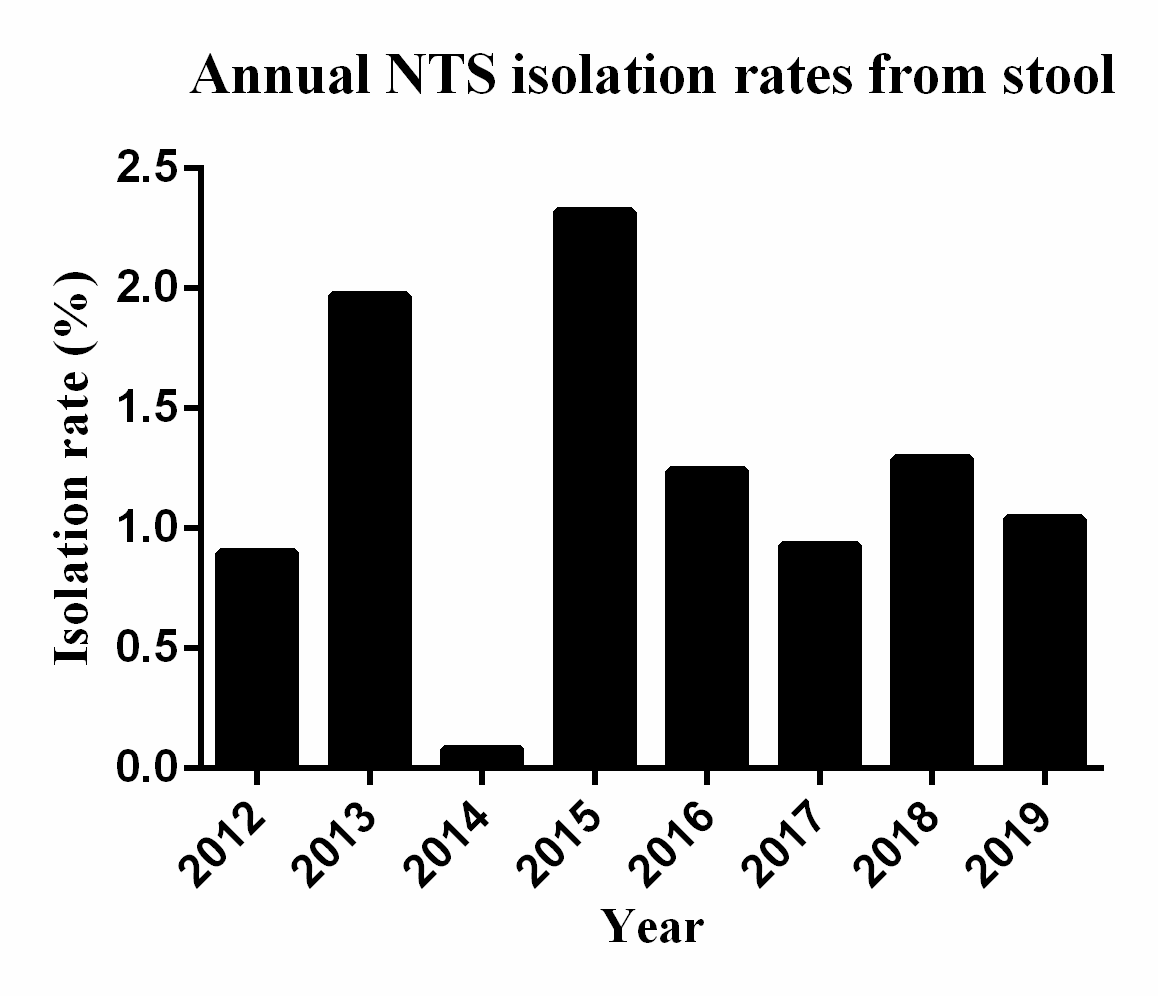

Supplement: S1 Fig — The bar chart shows the isolation rate of NTS isolates in each year from 2012 to 2019. (TIF) [file pntd.0008732.s001.tif]

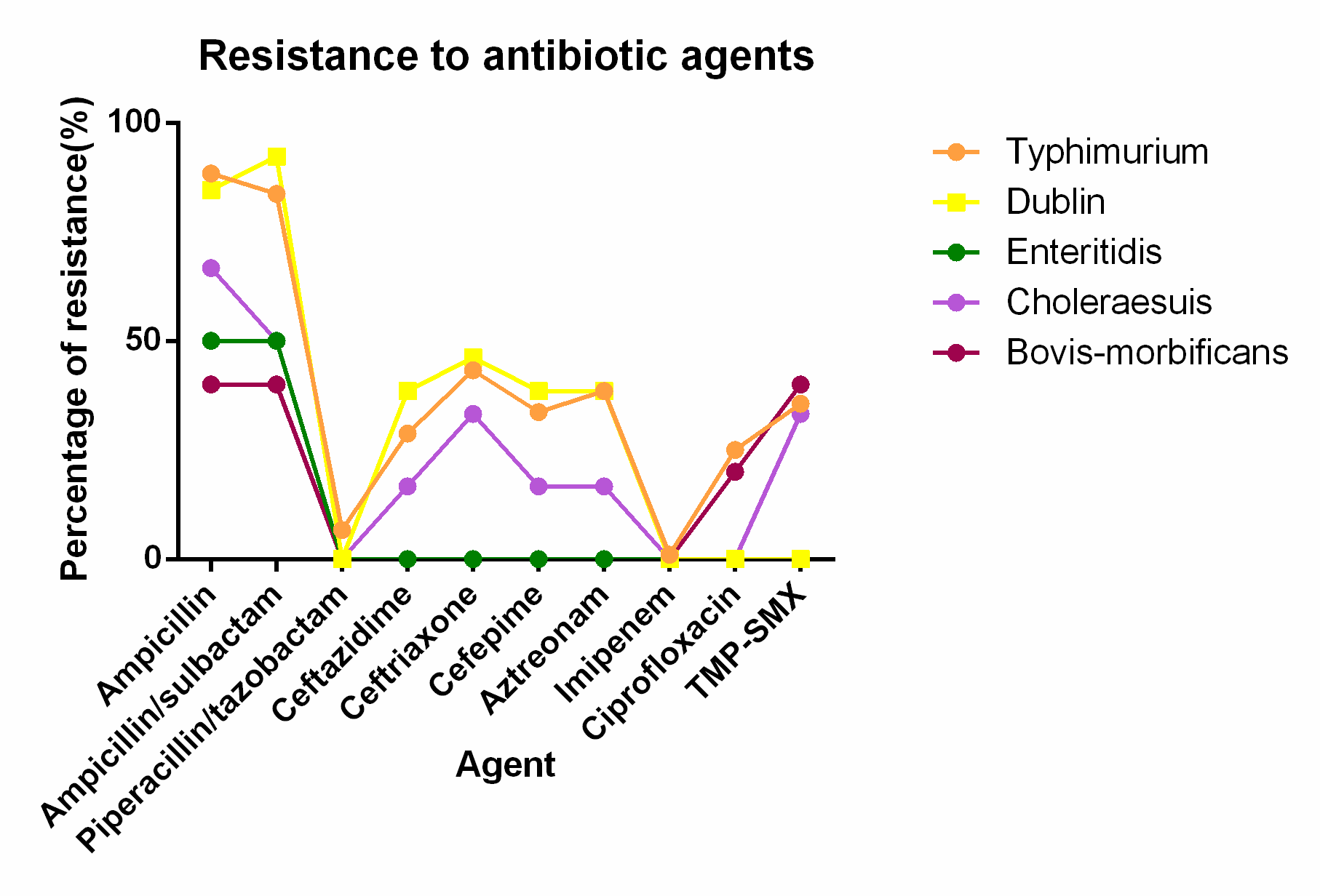

Supplement: S2 Fig — The percentage of resistance (%) over the 8-year study period in Ningbo is shown in the line graph. (TIF) [file pntd.0008732.s002.tif]

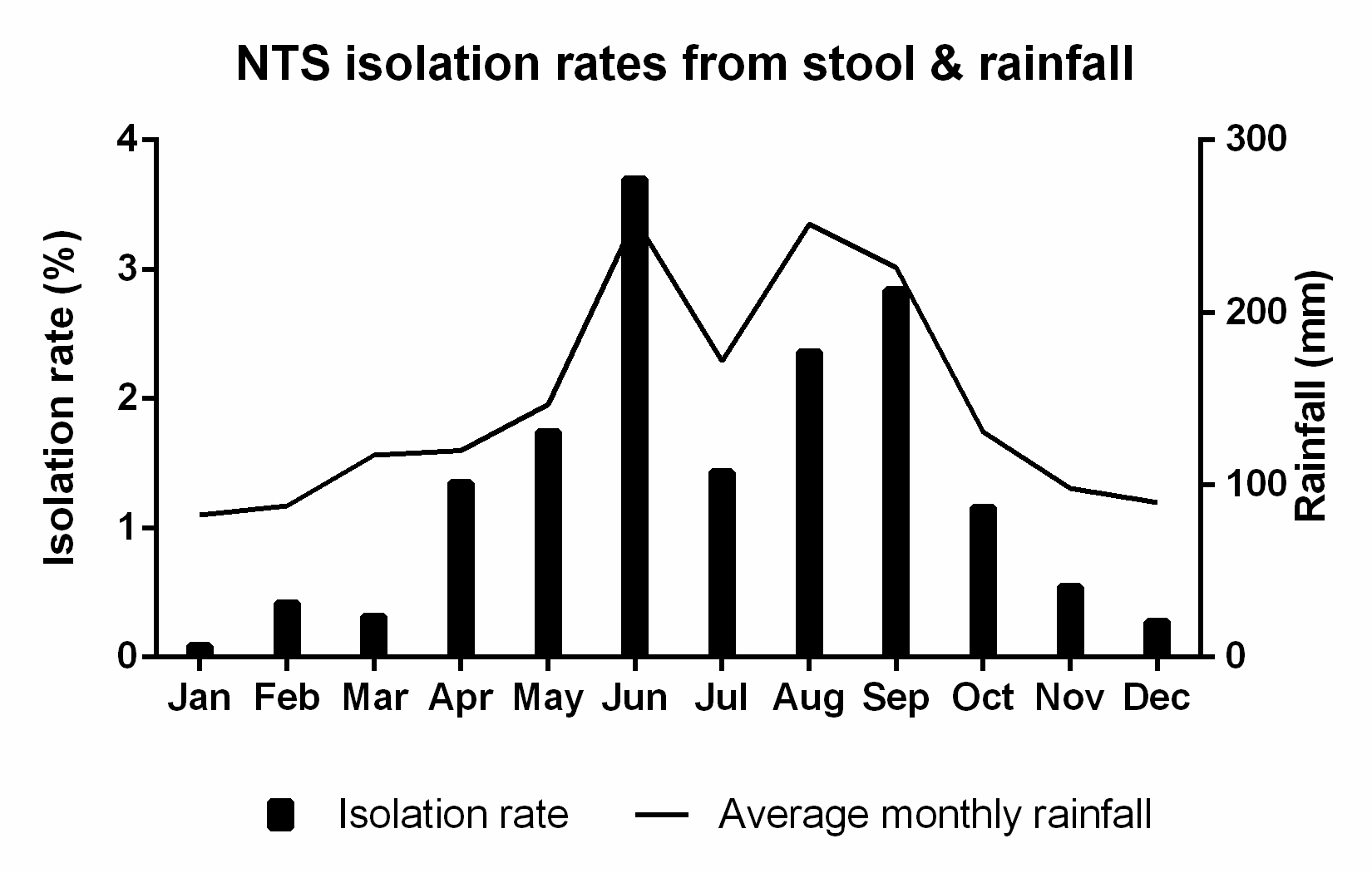

Supplement: S3 Fig — The bar chart shows the NTS isolation rates from stool according to the month. The average monthly rainfall over the 8-year study period in Ningbo is shown in the line graph. Abbreviations: NTS = non-typhoidal Salmonella, Jan = January, Feb = February, Mar = March, Apr = April, Jun = June, Jul = July, Aug = August, Sep = September, Oct = October, Nov = November, Dec = December. (TIF) [file pntd.0008732.s003.tif]

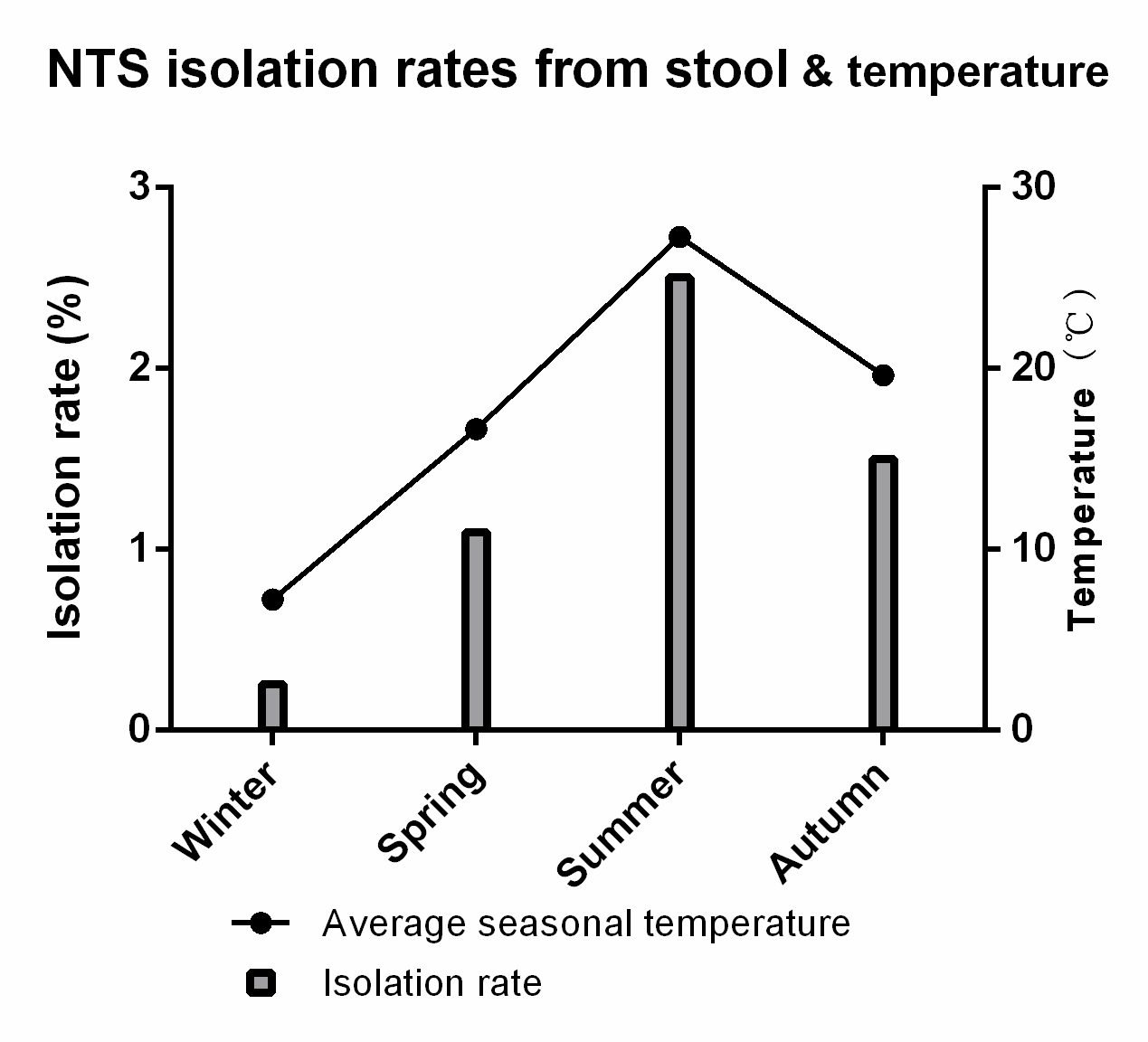

Supplement: S4 Fig — The bar chart shows the NTS isolation rates from stool. The average seasonal temperature over the 8-year study period in Ningbo is shown in the line graph. Abbreviations: NTS = non-typhoidal Salmonella. (TIF) [file pntd.0008732.s004.tif]
