# Supplementary material for: Non-typhoidal Salmonella infections among children in a tertiary hospital in Ningbo, Zhejiang, China, 2012–2019
Source: PLoS Negl Trop Dis. 2020 Oct 5;14(10):e0008732. doi: 10.1371/journal.pntd.0008732 (PMC7561262; doi:10.1371/journal.pntd.0008732)
Supplement: S1 Table — (DOCX) [file pntd.0008732.s005.docx]

**S1 Table. Details of demographic, isolation date, residence, serovar and clinical manifestations for each strain**

| No. | Gender | Age (months) | Isolation date | Rural location | Source | Serovar | Symptoms at admission | | | | | Co-morbidity | | |
| --- | --- | --- | --- | --- | --- | --- | --- | --- | --- | --- | --- | --- | --- | --- |
|  |  |  |  |  |  |  | Fever | Diarrhea | Bloody stools | Vomiting | Convulsion | Respiratory tract infection | Gastrointestinal virus infection | Leukemia |
| 1 | Male | 60 | 2012/1/2 | Y | Feces | *Other* | Y | Y | Y | Y | N | N | N | N |
| 2 | Female | 17 | 2012/2/21 | Y | Feces | *Other* | Y | Y | Y | N | N | Y | N | N |
| 3 | Female | 15 | 2012/4/5 | Y | Feces | Typhimurium | Y | Y | Y | N | Y | N | N | N |
| 4 | Female | 8 | 2012/4/13 | Y | Feces | Typhimurium | Y | Y | Y | Y | N | N | N | N |
| 5 | Male | 93 | 2012/4/29 | Y | Blood | Choleraesuis | Y | N | Y | Y | N | Y | N | Y |
| 6 | Male | 6 | 2012/6/8 | N | Feces | *Other* | Y | Y | Y | Y | N | Y | N | N |
| 7 | Female | 15 | 2012/6/19 | N | Feces | *Other* | N | Y | Y | N | N | N | N | N |
| 8 | Male | 8 | 2012/7/8 | Y | Feces | Typhimurium | Y | Y | Y | N | N | Y | N | N |
| 9 | Male | 8 | 2012/8/24 | Y | Feces | Typhimurium | Y | Y | Y | N | N | N | N | N |
| 10 | Male | 13 | 2012/9/8 | Y | Feces | Enteritidis | Y | Y | N | Y | N | N | N | N |
| 11 | Male | 14 | 2012/9/10 | N | Feces | *Other* | Y | Y | Y | Y | N | N | N | N |
| 12 | Male | <1 | 2012/9/18 | Y | Feces | *Other* | Y | Y | N | N | N | N | N | N |
| 13 | Male | 9 | 2012/9/22 | N | Feces | *Other* | Y | Y | Y | N | N | Y | N | N |
| 14* | Female | 1 | 2012/10/28 | N | Blood+CSF | *Other* | Y | N | N | Y | Y | Y | N | N |
| 15 | Female | 6 | 2012/11/15 | N | Blood | *Other* | Y | Y | Y | Y | N | N | N | N |
| 16 | Male | 14 | 2013/3/18 | N | Feces | Typhimurium | Y | Y | N | Y | Y | N | N | N |
| 17 | Male | 24 | 2013/3/23 | N | Feces | Enteritidis | Y | Y | Y | Y | Y | Y | Y | N |
| 18 | Male | 15 | 2013/4/13 | N | Feces | Typhimurium | Y | Y | Y | N | N | N | N | N |
| 19 | Female | 40 | 2013/4/14 | Y | Feces | Typhimurium | Y | Y | N | N | N | Y | N | N |
| 20 | Male | 12 | 2013/4/15 | N | Feces | Typhimurium | Y | Y | Y | N | N | N | N | N |
| 21 | Female | 26 | 2013/6/1 | N | Feces | Enteritidis | Y | Y | Y | Y | N | N | N | N |
| 22 | Male | 47 | 2013/6/1 | Y | Feces | Enteritidis | Y | Y | Y | Y | N | N | N | N |
| 23 | Male | 5 | 2013/6/2 | Y | Feces | Typhimurium | Y | Y | Y | N | N | Y | N | N |
| 24 | Male | 46 | 2013/6/3 | N | Feces | Enteritidis | N | Y | Y | Y | N | N | N | N |
| 25 | Male | 8 | 2013/6/11 | Y | Feces | Typhimurium | Y | Y | Y | Y | N | N | N | N |
| 26 | Male | 100 | 2013/6/14 | N | Feces | Typhimurium | N | N | Y | Y | N | N | N | N |
| 27* | Male | 31 | 2013/6/28 | N | Blood+Feces | Typhimurium | Y | Y | Y | N | N | Y | N | N |
| 28 | Female | 4 | 2013/7/15 | N | Feces | Typhimurium | Y | Y | Y | Y | N | N | N | N |
| 29 | Male | 8 | 2013/7/23 | Y | Feces | Typhimurium | Y | Y | Y | N | N | N | N | N |
| 30 | Female | 10 | 2013/7/24 | N | Feces | Typhimurium | Y | Y | Y | N | N | N | N | N |
| 31 | Female | 9 | 2013/7/25 | N | Feces | Typhimurium | Y | Y | Y | N | N | Y | Y | N |
| 32 | Male | 9 | 2013/7/31 | Y | Feces | Typhimurium | Y | Y | Y | Y | N | N | N | N |
| 33 | Female | 14 | 2013/8/7 | Y | Feces | Typhimurium | Y | Y | Y | N | N | Y | Y | N |
| 34 | Male | 15 | 2013/8/21 | N | Feces | Typhimurium | N | Y | N | Y | N | N | N | N |
| 35 | Female | 11 | 2013/8/22 | N | Feces | Enteritidis | Y | Y | Y | Y | N | N | N | N |
| 36 | Male | 21 | 2013/8/25 | N | Feces | Typhimurium | Y | Y | Y | Y | N | N | N | N |
| 37 | Female | 12 | 2013/8/29 | Y | Feces | Typhimurium | Y | Y | N | Y | N | N | N | N |
| 38 | Male | 2 | 2013/8/31 | Y | Feces | Typhimurium | Y | Y | N | N | N | N | N | N |
| 39 | Female | 16 | 2013/9/5 | Y | Feces | Typhimurium | Y | Y | Y | N | N | N | Y | N |
| 40 | Female | 6 | 2013/9/6 | N | Feces | Typhimurium | N | Y | Y | N | N | N | N | N |
| 41 | Female | 5 | 2013/9/6 | Y | Feces | Choleraesuis | Y | Y | Y | N | N | N | N | N |
| 42 | Female | 21 | 2013/9/7 | Y | Feces | Typhimurium | N | Y | N | Y | N | N | N | N |
| 43 | Female | 9 | 2013/9/7 | N | Feces | Typhimurium | Y | Y | Y | Y | N | N | N | N |
| 44 | Female | 14 | 2013/9/17 | Y | Feces | Typhimurium | Y | Y | Y | N | N | N | N | N |
| 45 | Female | 19 | 2013/9/20 | N | Feces | *Other* | N | Y | N | Y | N | N | N | N |
| 46 | Male | 49 | 2013/9/23 | Y | Feces | Typhimurium | Y | Y | Y | N | N | N | N | N |
| 47 | Female | 9 | 2013/10/5 | Y | Feces | Untyped | Y | Y | N | Y | Y | Y | N | N |
| 48 | Male | 13 | 2013/10/7 | N | Blood | Untyped | Y | Y | N | N | N | Y | N | N |
| 49 | Female | 9 | 2013/10/7 | N | Feces | Typhimurium | Y | Y | Y | N | N | N | N | N |
| 50 | Male | 20 | 2013/10/8 | N | Feces | Typhimurium | Y | Y | Y | N | N | Y | Y | N |
| 51 | Male | 6 | 2013/10/11 | Y | Feces | Typhimurium | Y | Y | Y | Y | N | Y | Y | N |
| 52 | Female | 13 | 2013/10/23 | Y | Feces | Typhimurium | N | N | Y | Y | N | N | N | N |
| 53 | Male | 9 | 2013/10/26 | Y | Feces | Typhimurium | Y | Y | Y | Y | N | N | Y | N |
| 54 | Female | 13 | 2013/11/6 | Y | Feces | Typhimurium | Y | Y | Y | N | N | Y | N | N |
| 55 | Male | 25 | 2013/11/30 | Y | Feces | Dublin | Y | Y | Y | Y | N | N | N | N |
| 56 | Female | 13 | 2013/12/1 | N | Feces | Dublin | Y | Y | N | Y | N | N | Y | N |
| 57 | Male | 46 | 2013/12/7 | N | Feces | Dublin | Y | Y | Y | N | N | N | N | N |
| 58 | Female | 1 | 2014/2/14 | N | Blood+CSF | Untyped | Y | N | N | N | N | N | Y | N |
| 59 | Male | 52 | 2014/3/3 | N | Feces | *Other* | N | Y | Y | N | N | N | N | N |
| 60 | Male | 6 | 2014/7/4 | Y | Blood | Untyped | Y | Y | Y | N | N | N | N | N |
| 61 | Male | <1 | 2014/10/10 | Y | Sputum | Dublin | N | N | Y | Y | Y | Y | N | N |
| 62 | Male | 5 | 2014/10/28 | N | Urine | *Other* | N | N | N | N | N | N | N | N |
| 63 | Male | 18 | 2015/4/23 | N | Feces | Typhimurium | Y | Y | Y | N | Y | N | N | N |
| 64 | Male | 6 | 2015/5/3 | Y | Feces | Enteritidis | N | N | Y | Y | N | N | N | N |
| 65 | Male | 11 | 2015/5/12 | Y | Feces | Untyped | Y | Y | N | Y | N | N | N | N |
| 66 | Female | 32 | 2015/5/12 | N | Feces | Choleraesuis | Y | Y | N | Y | Y | N | Y | N |
| 67 | Male | 6 | 2015/5/17 | N | Blood | Bovis-morbificans | N | Y | Y | N | N | N | N | N |
| 68 | Male | 9 | 2015/6/20 | N | Feces | Typhimurium | Y | Y | Y | N | N | N | Y | N |
| 69 | Female | 80 | 2015/6/26 | N | Feces | Dublin | Y | Y | Y | Y | N | Y | N | N |
| 70 | Male | 11 | 2015/6/30 | Y | Feces | Typhimurium | Y | Y | Y | Y | Y | N | N | N |
| 71 | Male | 20 | 2015/6/30 | N | Feces | Bovis-morbificans | Y | Y | N | Y | N | N | N | N |
| 72 | Male | 11 | 2015/7/1 | Y | Feces | Choleraesuis | N | Y | N | N | N | Y | N | N |
| 73 | Female | 12 | 2015/7/3 | N | Feces | Typhimurium | Y | Y | N | N | N | N | N | N |
| 74 | Female | 11 | 2015/7/5 | Y | Feces | Typhimurium | Y | Y | Y | N | N | N | N | N |
| 75 | Male | 4 | 2015/7/27 | Y | Feces | *Other* | Y | Y | Y | Y | N | N | N | N |
| 76 | Male | 9 | 2015/7/29 | N | Feces | Typhimurium | Y | Y | Y | N | N | N | N | N |
| 77 | Male | 24 | 2015/8/6 | N | Feces | Typhimurium | Y | Y | Y | Y | N | Y | N | N |
| 78 | Male | 9 | 2015/8/7 | N | Feces | Typhimurium | Y | Y | Y | N | N | N | N | N |
| 79 | Male | 5 | 2015/8/8 | N | Feces | Typhimurium | Y | Y | Y | N | N | Y | N | N |
| 80 | Female | 13 | 2015/8/19 | N | Feces | Typhimurium | Y | Y | Y | Y | N | N | Y | N |
| 81 | Male | 54 | 2015/8/28 | N | Feces | Typhimurium | Y | Y | Y | N | N | N | N | N |
| 82 | Male | 16 | 2015/9/4 | N | Feces | Typhimurium | Y | Y | N | N | N | N | N | N |
| 83 | Female | 6 | 2015/9/5 | Y | Feces | Typhimurium | N | Y | Y | N | N | N | N | N |
| 84 | Male | 10 | 2015/9/8 | Y | Feces | Typhimurium | Y | Y | N | Y | N | Y | N | N |
| 85 | Male | 52 | 2015/9/10 | Y | Feces | Typhimurium | Y | Y | Y | N | N | N | N | N |
| 86 | Female | 11 | 2015/9/23 | N | Feces | Typhimurium | Y | Y | Y | N | N | N | N | N |
| 87 | Male | 1 | 2015/9/28 | N | Feces | Typhimurium | N | Y | Y | N | N | N | N | N |
| 88 | Female | 44 | 2015/9/30 | N | Feces | *Other* | Y | Y | Y | N | Y | Y | N | N |
| 89 | Female | 14 | 2015/10/15 | N | Feces | Typhimurium | Y | Y | Y | Y | N | Y | N | N |
| 90 | Male | 13 | 2016/2/4 | N | Feces | Typhimurium | Y | Y | Y | N | Y | N | N | N |
| 91 | Female | 56 | 2016/4/19 | N | Feces | Typhimurium | Y | Y | Y | Y | N | N | N | N |
| 92 | Male | 32 | 2016/5/8 | N | Feces | Typhimurium | Y | Y | Y | N | N | N | N | N |
| 93 | Female | 15 | 2016/5/9 | N | Feces | Choleraesuis | Y | Y | N | N | N | N | Y | N |
| 94 | Male | 7 | 2016/5/16 | N | Feces | Typhimurium | Y | Y | Y | N | N | N | Y | N |
| 95 | Male | 46 | 2016/6/22 | N | Feces | Dublin | Y | Y | N | Y | N | Y | N | N |
| 96 | Male | 16 | 2016/6/23 | N | Feces | Dublin | Y | Y | Y | Y | N | Y | Y | N |
| 97 | Male | <1 | 2016/6/28 | N | Sputum | Dublin | N | N | Y | N | N | Y | N | N |
| 98 | Female | 26 | 2016/8/7 | N | Feces | *Other* | Y | Y | Y | Y | N | Y | N | N |
| 99 | Male | 19 | 2016/8/8 | Y | Feces | Typhimurium | Y | Y | Y | N | N | N | N | N |
| 100 | Male | 5 | 2016/8/16 | N | Feces | Typhimurium | Y | Y | Y | N | N | N | N | N |
| 101 | Male | 9 | 2016/9/6 | Y | Feces | Typhimurium | Y | Y | N | N | N | N | N | N |
| 102 | Male | 106 | 2016/10/8 | Y | CSF | Enteritidis | Y | N | N | Y | Y | N | N | Y |
| 103 | Male | 3 | 2016/10/10 | Y | Feces | Bovis-morbificans | Y | Y | Y | N | N | N | N | N |
| 104 | Male | 15 | 2016/12/3 | N | Feces | Typhimurium | N | Y | N | N | Y | N | N | N |
| 105 | Female | 116 | 2016/12/3 | N | Feces | Typhimurium | Y | Y | Y | Y | N | N | N | N |
| 106 | Male | 30 | 2017/4/7 | N | Feces | Typhimurium | Y | Y | Y | N | Y | N | N | N |
| 107 | Female | 118 | 2017/5/25 | N | Feces | Typhimurium | Y | Y | Y | Y | N | N | N | N |
| 108 | Male | 8 | 2017/5/26 | N | Feces | Typhimurium | Y | Y | Y | N | N | N | Y | N |
| 109 | Male | 16 | 2017/6/1 | N | Feces | Typhimurium | Y | Y | Y | N | N | N | N | Y |
| 110 | Female | 14 | 2017/6/11 | N | Feces | Typhimurium | Y | Y | Y | Y | N | N | N | N |
| 111 | Male | 16 | 2017/6/14 | Y | Feces | Typhimurium | Y | Y | Y | Y | N | Y | N | N |
| 112 | Male | 7 | 2017/6/22 | N | Feces | Typhimurium | Y | Y | Y | N | N | Y | Y | N |
| 113 | Male | 16 | 2017/8/7 | N | Feces | *Other* | Y | Y | Y | Y | N | Y | N | N |
| 114 | Female | 4 | 2017/8/31 | Y | Feces | Typhimurium | Y | Y | Y | N | N | N | N | N |
| 115 | Male | 14 | 2017/9/15 | N | Feces | Other | N | Y | N | N | N | N | N | N |
| 116 | Male | 10 | 2017/9/23 | N | Feces | Typhimurium | Y | Y | Y | Y | N | Y | N | N |
| 117 | Female | 2 | 2017/10/3 | N | Feces | Typhimurium | Y | Y | Y | N | N | N | N | N |
| 118 | Female | 7 | 2017/10/22 | N | Feces | Typhimurium | Y | Y | N | Y | N | N | N | N |
| 119 | Female | 2 | 2017/12/6 | N | Feces | Typhimurium | N | Y | Y | Y | N | N | N | N |
| 120 | Male | 15 | 2018/4/28 | Y | Feces | Typhimurium | Y | Y | N | N | N | N | N | N |
| 121 | Female | 1 | 2018/5/16 | N | Feces | Typhimurium | Y | Y | Y | N | N | N | N | N |
| 122 | Male | 1 | 2018/5/18 | N | Feces | Typhimurium | Y | Y | Y | Y | N | Y | N | N |
| 123 | Female | 29 | 2018/5/26 | N | Feces | Dublin | Y | N | Y | N | Y | N | N | N |
| 124 | Male | 83 | 2018/5/29 | N | Feces | Bovis-morbificans | Y | Y | Y | N | N | N | N | N |
| 125 | Male | 20 | 2018/6/15 | N | Feces | Typhimurium | Y | Y | Y | N | N | N | N | N |
| 126 | Female | 33 | 2018/6/24 | N | Feces | Typhimurium | Y | Y | Y | N | N | Y | N | N |
| 127 | Female | 10 | 2018/6/30 | Y | Feces | Typhimurium | Y | Y | Y | Y | N | N | N | N |
| 128 | Female | 18 | 2018/6/30 | N | Feces | *Other* | Y | Y | N | Y | N | Y | N | N |
| 129 | Male | 20 | 2018/7/19 | N | Feces | Typhimurium | Y | Y | Y | Y | N | Y | N | N |
| 130 | Female | 11 | 2018/8/11 | Y | Feces | *Other* | Y | Y | N | N | N | N | N | N |
| 131 | Male | 9 | 2018/8/17 | N | Feces | Typhimurium | Y | Y | N | Y | N | Y | N | N |
| 132 | Male | 8 | 2018/8/19 | Y | Feces | *Other* | Y | Y | Y | N | N | N | N | N |
| 133 | Male | 12 | 2018/8/23 | N | Feces | Typhimurium | Y | Y | Y | N | N | N | N | N |
| 134 | Male | 67 | 2018/8/26 | Y | Feces | Typhimurium | Y | Y | N | N | N | Y | N | N |
| 135 | Male | 11 | 2018/8/28 | Y | Feces | Dublin | N | Y | Y | N | N | N | N | N |
| 136 | Male | 7 | 2018/9/7 | Y | Feces | Choleraesuis | Y | Y | Y | N | N | N | N | N |
| 137 | Female | 8 | 2018/9/10 | N | Feces | Bovis-morbificans | Y | Y | Y | N | N | N | N | N |
| 138 | Male | 6 | 2018/9/22 | Y | Feces | Typhimurium | Y | Y | Y | N | N | N | N | N |
| 139 | Male | 11 | 2018/10/5 | Y | Feces | *Other* | Y | Y | Y | Y | N | N | N | N |
| 140 | Female | 15 | 2018/10/8 | Y | Feces | Typhimurium | Y | Y | Y | N | N | N | N | N |
| 141 | Male | 13 | 2018/10/18 | N | Feces | Typhimurium | Y | Y | Y | N | N | N | N | N |
| 142 | Female | 28 | 2018/11/5 | Y | Feces | Typhimurium | Y | Y | Y | Y | N | N | N | N |
| 143 | Male | 7 | 2019/2/21 | N | Feces | Dublin | Y | Y | Y | Y | N | Y | N | N |
| 144 | Female | 28 | 2019/4/1 | N | Feces | Dublin | Y | Y | Y | N | N | N | N | N |
| 145 | Male | 103 | 2019/4/19 | N | Feces | *Other* | Y | Y | Y | Y | N | N | N | N |
| 146 | Female | 21 | 2019/5/25 | Y | Feces | Typhimurium | Y | Y | Y | N | N | N | Y | N |
| 147 | Male | 17 | 2019/5/31 | N | Feces | Typhimurium | Y | Y | Y | N | Y | N | N | N |
| 148 | Male | 3 | 2019/6/5 | N | Feces | Typhimurium | Y | Y | Y | N | N | N | Y | N |
| 149 | Female | 23 | 2019/6/8 | N | Blood | Typhimurium | Y | Y | Y | Y | N | N | N | N |
| 150 | Female | 8 | 2019/6/17 | N | Feces | *Other* | N | Y | Y | N | N | N | N | N |
| 151 | Female | 15 | 2019/6/21 | Y | Feces | Typhimurium | Y | Y | Y | N | N | Y | N | N |
| 152 | Male | 18 | 2019/6/25 | Y | Feces | Typhimurium | Y | Y | Y | N | N | N | N | N |
| 153 | Male | 40 | 2019/7/2 | N | Feces | Typhimurium | Y | Y | Y | N | N | N | N | N |
| ·154 | Female | 13 | 2019/7/3 | Y | Feces | Typhimurium | Y | Y | N | N | N | N | N | N |
| 155 | Male | 13 | 2019/7/15 | N | Feces | Typhimurium | Y | Y | Y | N | N | Y | N | N |
| 156 | Female | 1 | 2019/7/28 | Y | Feces | Typhimurium | Y | Y | Y | N | N | Y | N | N |
| 157 | Male | 15 | 2019/9/2 | Y | Feces | Typhimurium | Y | Y | Y | N | N | N | N | N |
| 158 | Female | 13 | 2019/9/12 | Y | Feces | Typhimurium | Y | Y | N | N | N | Y | N | N |
| 159 | Male | 15 | 2019/9/22 | N | Feces | Typhimurium | Y | N | Y | N | N | Y | N | N |
| 160 | Female | 23 | 2019/9/27 | N | Feces | Typhimurium | Y | N | N | N | N | Y | N | N |
| 161 | Female | 23 | 2019/10/1 | N | Feces | Typhimurium | Y | N | N | N | N | Y | N | N |
| 162 | Female | 12 | 2019/10/13 | N | Feces | Typhimurium | Y | N | Y | N | Y | Y | N | N |
| 163 | Male | 5 | 2019/11/4 | Y | Feces | Typhimurium | Y | Y | Y | N | N | N | N | N |
| 164 | Male | 162 | 2019/11/6 | N | Blood | Dublin | N | N | N | Y | N | Y | N | N |
| 165 | Male | 50 | 2019/11/26 | N | Feces | *Other* | Y | Y | N | Y | N | Y | Y | N |
| 166 | Male | 22 | 2019/12/17 | Y | Feces | *Other* | Y | Y | Y | Y | N | Y | N | N |
| * Refer to the case presented clinically as meningitis. CSF: cerebrospinal fluid; N: no; Y: yes. | | | | | | | | | | | | | | |
